# Genomic insight into strategy, interaction and evolution of nitrifiers in metabolizing key labile-dissolved organic nitrogen in different environmental niches

**DOI:** 10.3389/fmicb.2023.1273211

**Published:** 2023-12-13

**Authors:** Qian Liu, Yuhao Chen, Xue-Wei Xu

**Affiliations:** ^1^Donghai Laboratory, Zhoushan, Zhejiang, China; ^2^Key Laboratory of Marine Ecosystem Dynamics, Second Institute of Oceanography, Ministry of Natural Resources, Hangzhou, Zhejiang, China; ^3^Ocean College, Zhejiang University, Hangzhou, Zhejiang, China; ^4^School of Oceanography, Shanghai Jiao Tong University, Shanghai, China

**Keywords:** ammonia-oxidizing archaea, ammonia-oxidizing bacteria, nitrite-oxidizing bacteria, comammox, dissolved organic nitrogen, urea, polyamine, cyanate

## Abstract

Ammonia-oxidizing archaea (AOA) and bacteria (AOB), nitrite-oxidizing bacteria (NOB), and complete ammonia oxidizers (comammox) are responsible for nitrification in nature; however, some groups have been reported to utilize labile-dissolved organic nitrogen (LDON) for satisfying nitrogen demands. To understand the universality of their capacity of LDON metabolism, we collected 70 complete genomes of AOA, AOB, NOB, and comammox from typical environments for exploring their potentials in the metabolism of representative LDON (urea, polyamines, cyanate, taurine, glycine betaine, and methylamine). Genomic analyses showed that urea was the most popular LDON used by nitrifiers. Each group harbored unique urea transporter genes (AOA: *dur3* and *utp*, AOB: *utp*, and NOB and comammox: *urtABCDE* and *utp*) accompanied by urease genes *ureABC*. The differentiation in the substrate affinity of these transporters implied the divergence of urea utilization efficiency in nitrifiers, potentially driving them into different niches. The cyanate transporter (*cynABD* and *focA/nirC*) and degradation (*cynS*) genes were detected mostly in NOB, indicating their preference for a wide range of nitrogen substrates to satisfy high nitrogen demands. The lack of genes involved in the metabolism of polyamines, taurine, glycine betaine, and methylamines in most of nitrifiers suggested that they were not able to serve as a source of ammonium, only if they were degraded or oxidized extracellularly as previously reported. The phylogenetic analyses assisted with comparisons of GC% and the Codon Adaptation Index between target genes and whole genomes of nitrifiers implied that urea metabolic genes *dur3* and *ureC* in AOA evolved independently from bacteria during the transition from *Thaumarchaeota* to AOA, while *utp* in terrestrial AOA was acquired from bacteria via lateral gene transfer (LGT). Cyanate transporter genes *cynS* and *focA*/*nirC* detected only in a terrestrial AOA *Candidadus* Nitrsosphaera gargensis Ga9.2 could be gained synchronously with *Nitrospira* of NOB by an ancient LGT. Our results indicated that LDON utilization was a common feature in nitrifiers, but metabolic potentials were different among nitrifiers, possibly being intensely interacted with their niches, survival strategies, and evolutions.

## 1 Introduction

Labile-dissolved organic nitrogen (LDON) compounds are the groups with low-molecule weights and rapid turnover rates in the environments (Sipler et al., 2013). They are generally produced from the degradation of proteins or released from primary producers (e.g., phytoplankton in the ocean), and preferentially taken up by heterotrophic bacteria as nitrogen sources (e.g., Liu et al., 2016, 2022b; Damashek et al., 2019). LDON compounds generally include dissolved free amino acids, urea, polyamines, methylamines, taurine, cyanate, and glycine betaine. Their uptake contributes significantly to bacterial nitrogen demands and enhances nitrogen cycling (e.g., Jørgensen, 2006; Liu et al., 2015, 2022a; Clifford et al., 2019). In addition, photoautotrophic phytoplankton in the ocean can also assimilate or oxidize LDON for acquiring nitrogen and energy, especially in N-limiting environments (Glibert et al., 2016). In recent years, more studies reveal that the chemoautotrophic prokaryotes involved in nitrification (e.g., *Nitrospinae* and *Thaumarchaeota*) may be capable of utilizing LDON for enhancing or sustaining the growth as well (Koch et al., 2015; Qin et al., 2017; Kitzinger et al., 2019, 2020).

The nitrification process is one of the most important steps in the nitrogen cycle driven by a complex microbial consortium (Voss et al., 2013), including ammonia-oxidizing archaea (AOA) and bacteria (AOB), nitrite-oxidizing bacteria (NOB), and complete ammonia oxidizer (comammox; He et al., 2018). They are key players in global nitrogen and carbon cycles (Bayer et al., 2019). AOA and AOB perform ammonia oxidation, the first and rate-limiting step of nitrification (Könneke et al., 2005), and NOB catalyze the second step of nitrification by oxidizing nitrite to nitrate (Daims et al., 2016). Comammox are capable of converting ammonia to nitrate in one step (Hu and He, 2017). AOA usually outcompete AOB for ammonia and play a major role in controlling ammonia oxidation in most environments due to their relatively higher affinity for ammonia (Stahl and de la Torre, 2012). They are mainly categorized into four phylogenetic lineages, namely, *Nitrosopumilales* (Group I.1a), “*Ca*. Nitrosotaleales” (Group I.1a-associated), *Nitrososphaerales* (Group I.1b), and “*Ca*. Nitrosocaldales” (Jung et al., 2022). AOB are commonly detected in ammonia-rich environments, such as sewage treatment plants, eutrophic freshwater, coastal waters, and soil (Soliman and Eldyasti, 2018). A total of five genera have been identified as AOB, in which *Nitrosomonas*, *Nitrosospira*, *Nitrosovibrio*, and *Nitrosolobus* belong to the subclass β-Proteobacteria and *Nitrosococcus* to the subclass γ-Proteobacteria. Among all known NOB, the genus *Nitrospira* appears to be most widespread and phylogenetically diverse in different habitats (Koch et al., 2015). *Nitrospira* strains are well adapted to low nitrite concentrations and form at least six phylogenetic lineages that are globally distributed in soils, oceans, freshwater, hot springs, etc. (Koch et al., 2015). *Nitrospinae* are the dominant marine NOB and can reach high abundances (up to ∼10% of the microbial community) in mesopelagic zones, oxygen minimum zones (OMZs), deep-sea waters, and sediments (Daims et al., 2016). The comammox *Nitrospira* are abundant in natural and engineered habitats. It is reported that comammox may functionally outcompete other canonical nitrifiers under highly oligotrophic conditions (Hu and He, 2017).

The capability of utilizing extracellular LDON may increase nitrogen assimilation and be beneficial for the production of energy and biomass of nitrifiers (Kitzinger et al., 2019). Ammonia oxidizers (AOM) have been suggested to utilize extracellular LDON as an alternative source of ammonia under the situation of ammonia limitation (Sliekers et al., 2004; Qin et al., 2017), while NOB use them for reciprocal feeding with AOM (Palatinszky et al., 2015). The urea utilization has been detected in verified experiments for AOA strains *Ca*. Nitrososphaera gargensis Ga9.2, *N*. *viennensis* EN76, and *Nitrosopumilus ureiphilus* PS0 (Wetzel et al., 2011; Qin et al., 2014; Damashek et al., 2019), AOB strains *Nitrosomonas oligotropha* and *N*. *ureae* (Tourna et al., 2011; Spang et al., 2012; Qin et al., 2014), and NOB *Nitrospira moscoviensis* (Sliekers et al., 2004). The field samples collected from marine environments also reveal that AOA and *Nitrospinae* of NOB can incorporate urea-and cyanate-derived nitrogen at significantly higher rates than other microorganisms (Kitzinger et al., 2018, 2020). A recent study in the Gulf of Mexico found that AOA mainly used ammonium, while most of the cellular nitrogen-demand of *Nitrospinae* was met by the assimilation of urea and cyanate (Kitzinger et al., 2020). The alternative utilization of LDON for avoiding the competition with ammonia-oxidizing microbes may be a key factor for ecological success of NOB (Kitzinger et al., 2020). Moreover, the metagenome-assembled genomes (MAGs) of *Nitrospinae* encode ABC-type transporter of spermidine, amino acids, and peptides, an indication for their additional nitrogen sources for growth (Kitzinger et al., 2020). Therefore, the potential of nitrifiers in utilizing LDON could be related to their survival strategies.

Although a few studies have showed that nitrifying microbes are capable of utilizing LDON based on both laboratory experiments or genomic analysis (Tourna et al., 2011; Palatinszky et al., 2015; Qin et al., 2017), as more strains are identified from different habitats, little has been done to systematically catalog the metabolic potential of LDON of these nitrifiers from different environments for understanding their utilization mechanisms and strategies. Whether it is a common metabolic process or only occurs in certain environments needs more investigation. Moreover, since the availability of LDON increases rapidly as a consequence of anthropogenic impact, especially in estuary and coastal waters (Seitzinger et al., 2002), assessing the potentials of nitrifiers in the utilization of LDON can further explore the ecological role of LDON in the ecosystem. To fill this gap, we compared metabolic potentials of LDON among AOA, AOB, NOB, and comammox, and between marine and terrestrial taxa based on genomic analyses, to discuss mechanisms and strategies of LDON utilization by nitrifiers in different environments.

## 2 Materials and methods

### 2.1 Genomic information collection

The complete genome sequences of representative AOA (*n* = 46), AOB (*n* = 10), NOB (*n* = 12), and comammox (*n* = 2) strains were collected from National Center for Biotechnology Information (NCBI; https://www.ncbi.nlm.nih.gov/), Joint Genome Institute (JGI; https://jgi.doe.gov/), or Beijing Institute of Genomics Data Center (BIGD; https://ngdc.cncb.ac.cn/) according to the accession number (Supplementary Table S1). The dataset included all available genomes from isolated and enriched AOA strains, representatives of available genomes of AOB and NOB, and selected metagenomic-assembled genomes (MAGs, completeness >70%, low completeness could result in non-detection of target genes) of nitrifiers from extreme marine environments (Supplementary Table S1). Diverse habitats from both marine (e.g., sediment, estuary, coastal seawater, and deep-sea) and terrestrial (e.g., soil, freshwater, and wastewater treatment plant) environments were covered for the subsequent analysis and comparison (Supplementary Table S1).

### 2.2 Cell volume estimation

We recorded the shape, width, and length of cells for collected AOA, AOB, NOB, and comammox according to the description in the literature (Supplementary Table S2). The ratio of surface area and cell volume (SA/V) was estimated assuming a spherical cell based on equation (1) (Pachiadaki et al., 2017). The SA/V ratio of a rod-shaped cell was calculated with equation (2) (Boyde and Williams, 1971). Cell widths and lengths used in the formula were means of values in corresponding references (Supplementary Table S2).


(1) $$\frac{S}{V}=\frac{\pi \times d^{2}}{\frac{4}{3}\times \pi \times {\left(\frac{d}{2}\right)}^{3}}$$

(2) $$\frac{SA}{V}=\frac{\pi \times d\times h+2\times \pi \times {\left(\frac{d}{2}\right)}^{2}}{\pi \times {\left(\frac{d}{2}\right)}^{2}\times h}$$

where *V* is the cell volume, *d* is the cell diameter, *h* is the cell length, and *SA* is the surface area.

### 2.3 Gene collection and genome annotation

**Figure 1:**
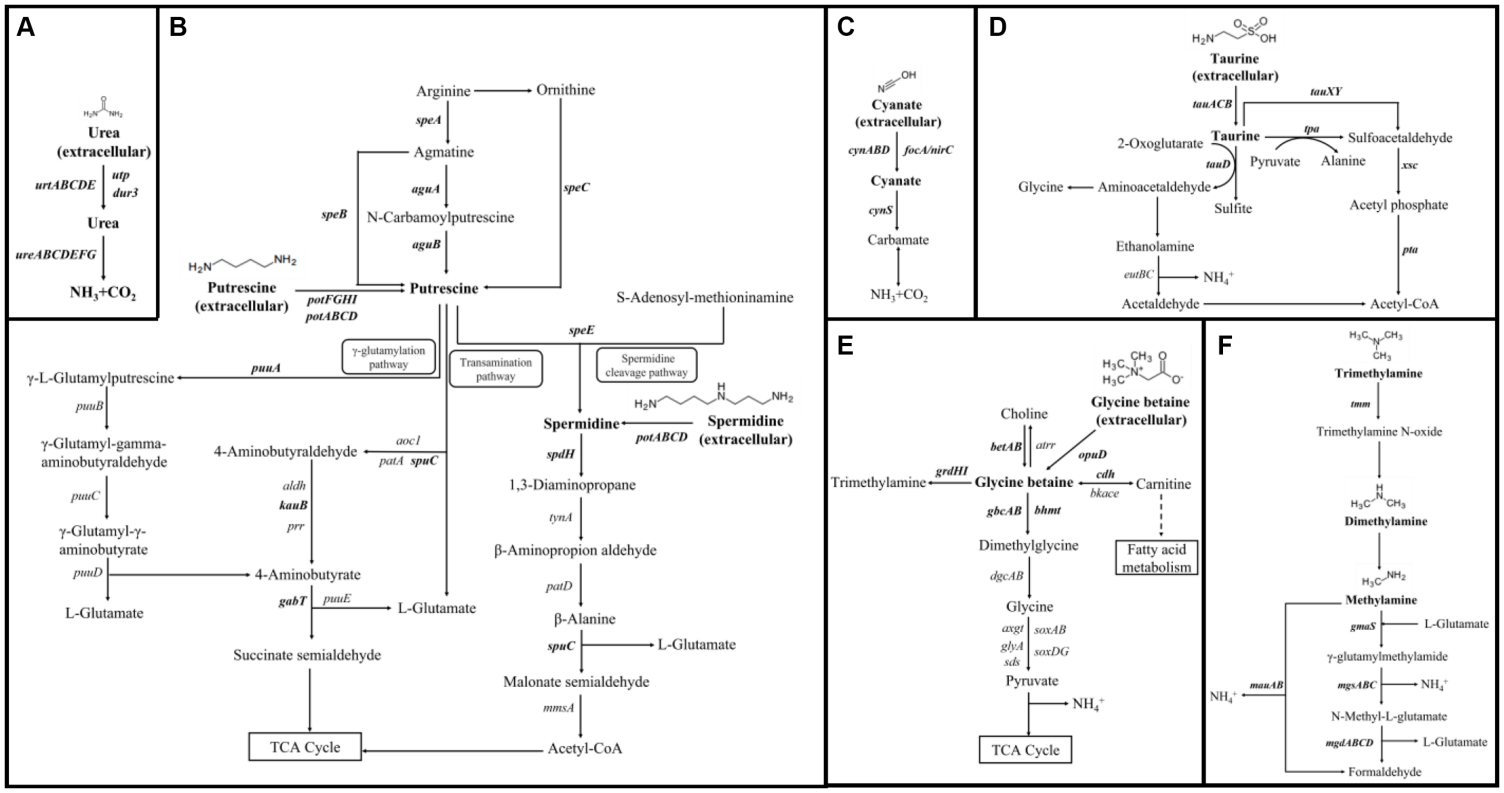
Metabolic pathways and products of representative labile-dissolved organic nitrogen (LDON), including **(A)** urea (Spang et al., 2012; Veaudor et al., 2019), **(B)** polyamines (Igarashi and Kashiwagi, 2010; Mou et al., 2011), **(C)** cyanate (Maeda and Omata, 2009; Palatinszky et al., 2015), **(D)** taurine (Cook and Denger, 2006; Engelberts et al., 2020; Rohwerder, 2020), **(E)** glycine betaine (Li et al., 2021; Boysen et al., 2022), and **(F)** methylamine (Chen, 2012; Taubert et al., 2017). Gene involved in metabolic processes are listed in italics. The LDON compounds and genes investigated in this study are highlighted in bold. Black dotted arrows represent the potential fates of molecules.

Amino acid sequences of key functional genes related to ammonia oxidation, and transport, biosynthesis, and degradation of selected LDON compounds (urea, polyamines, cyanate, taurine, glycine betaine, and methylamines) were collected from the NCBI (Figure 1; Supplementary Table S3) for the subsequent alignment with genomes of AOA, AOB, NOB, and comammox strains. They included genes encoding ammonia monooxygenase (*amoABC*, K10944-K10946; Zhang et al., 2023), bacteria-type urea ABC transporter (*urtABCDE*, K11959-K11963; Veaudor et al., 2019), a prokaryote-origin mammalian urea transporter (*utp*, K08717; Minocha et al., 2003; Levin et al., 2009; Spang et al., 2012), urea active transporter (*dur3*, K20989) generally detected in marine unicellular photosynthetic eukaryotes (Solomon et al., 2010), urease (*ureABC*, K01428-K01430; *ureDFG*, K03188-K03190; *ureE*, K03187; Veaudor et al., 2019), polyamine ABC transporter (spermidine-preferential ABC transporter, *potABCD*, K11069-K11072; putrescine ABC transporter, *potFGHI*, K11073-K11076; Mou et al., 2010), cyanate ABC transporter (*cynABD*, K15576, K15577, and K15579; Maeda and Omata, 2009), a formate–nitrite transporter (FNT, *focA*/*nirC*, K21990) functioning in cyanate assimilation in cyanobacteria (Maeda and Omata, 2009), cyanase (*cynS*, K01725; Taubert et al., 2017), taurine ABC transporter (*tauACB*, K15551, K15552, and K10831; Cook and Denger, 2006; Rohwerder, 2020), glycine betaine transporter (*opuD*, K05020; Wetzel et al., 2011; Boysen et al., 2022), trimethylamine (TMA) monooxygenase (*tmm*, K18277), glutamate-methylamine (GMA) synthetase (*gmaS*, K01949), N-Methyl-L-glutamate (NMG) synthase (*mgsABC*, K22081-K22083), NMG dehydrogenase (*mgdABCD*, K22084-K22087), methylamine dehydrogenase (*mauAB*, K15228, K15229) in methylamines metabolism (Chen, 2012; Taubert et al., 2017), and enzymes involved in polyamine biosynthesis (*speA*, K01585; *speB*, K01480; *speC*, K01581; *speE*, K00797; *aguA*, K10536; *aguB*, K12251) and catabolism (*puuA*, K09470; *spuC*, K12256; *kauB*, K12254; *gabT*, K07250; *spdH*, K00316; Mou et al., 2010, 2011), taurine degradation (*tauD*, K03119; *tpa*, K03851; *xsc*, K03852; *pta*, K13788; *tauXY*, K07255 and K07256; Cook and Denger, 2006; Rohwerder, 2020), glycine betaine synthesis (*betAB*, K00108, and K00130), and catabolism (*gbcAB*, K00479, and K21832; *bhmt*, K00544; *grdHI*, K21579, and K21578; *cdh*, K17735; Wetzel et al., 2011; Boysen et al., 2022). The genome-wide gene annotation was carried out through the website of Rapid Annotation using Subsystem Technology (RAST, https://rast.nmpdr.org/) with all collected genomes of AOA, AOB, NOB, and comammox. RAST annotation results and the NCBI database were used to identify homologs of targeted genes (Supplementary Table S3). All functional gene sequences were subjected to BLASTp. The amino acid sequence with a percent identity to the reference gene (Supplementary Table S3) greater than 40% was considered to be the homolog of the target gene (Pearson, 2013).

### 2.4 Phylogenetic analysis of key functional genes

To demonstrate the evolution of functional genes involved in LDON metabolism, phylogenetic trees were constructed with amino acid sequences for key genes by maximum likelihood based on the model of Le and Gascuel (2008) with 1,000 bootstrap replications using the software MEGA 7.0 (Kumar et al., 2016). The amino acid sequences were aligned using Clustal W (Thompson et al., 1994). The best model was used after the alignment (Hall, 2013). Models with the lowest Bayesian information criterion (BIC) score were considered to best describe the substitution pattern (Bast, 2013). A discrete gamma distribution was used to model differences of evolutionary rates among sites [five categories (+G)]. For *utp* and *focA*/*nirC*, the rate variation model allowed for some sites to be evolutionarily invariable ([+I]). All phylogenetic trees were drawn to scale with branch lengths measured in the number of substitutions per site.

The GC contents of key genes of selected AOA, AOB, NOB, and comammox, and outgroup species in phylogenetic analyses were calculated using GC Content Calculator,^1^ while those of whole genomes were obtained from the NCBI database.^2^ The values of the Codon Adaptation Index (CAI) of the same group of genes and whole genomes of selected nitrifiers and outgroup species were calculated using an online CAI calculator.^3^

### 2.5 Data and material availability

Genome sequence data are available in NCBI, JGI, or BIGD databases, and their accession numbers are listed in Supplementary Table S1. All other data products associated with this study are available from the corresponding authors upon request.

## 3 Results

### 3.1 Genomic and phenotypic characteristics of collected nitrifiers

Collected strains of AOA belonging to the genera *Nitrosopumilus*, *Nitrosopelagicus*, *Nitrosomarinus*, and *Cenarchaeum* were all of marine origin (*n* = 26). The genera *Nitrosarchaeum*, *Nitrosotenuis*, *Nitrosotalea*, *Nitrososphaera*, *Nitrosocosmicus*, and *Nitrosocaldus* were mostly from terrestrial environments, including hot springs, lakes, and soil (*n* = 20; Supplementary Table S1). Only strains *Ca*. Nitrosarchaeum limnium SFB1 and BG20 were enriched from marine environments (Supplementary Table S1). In AOB, all three *Nitrosococcus* strains were enriched from marine environments, and *Nitrosomonas* strains and *Ca*. Nitrosacidococcus tergens sp. RJ19 were terrestrial (Supplementary Table S1). *Nitrospina gracilis*, *Nitrospira marina* Nb-295, *Ca*. Nitrohelix vancouverensis, and *Ca*. Nitronauta litoralis of NOB were inhabited in marine environments, and two *Nitrobacter* strains, *Ca*. Nitrotoga arctica, and the rest of *Nitrospira* species including two comammox *Ca*. N. inopinata and *Ca*. N. kreftii were terrestrial origin (Supplementary Table S1).

**Figure 2:**
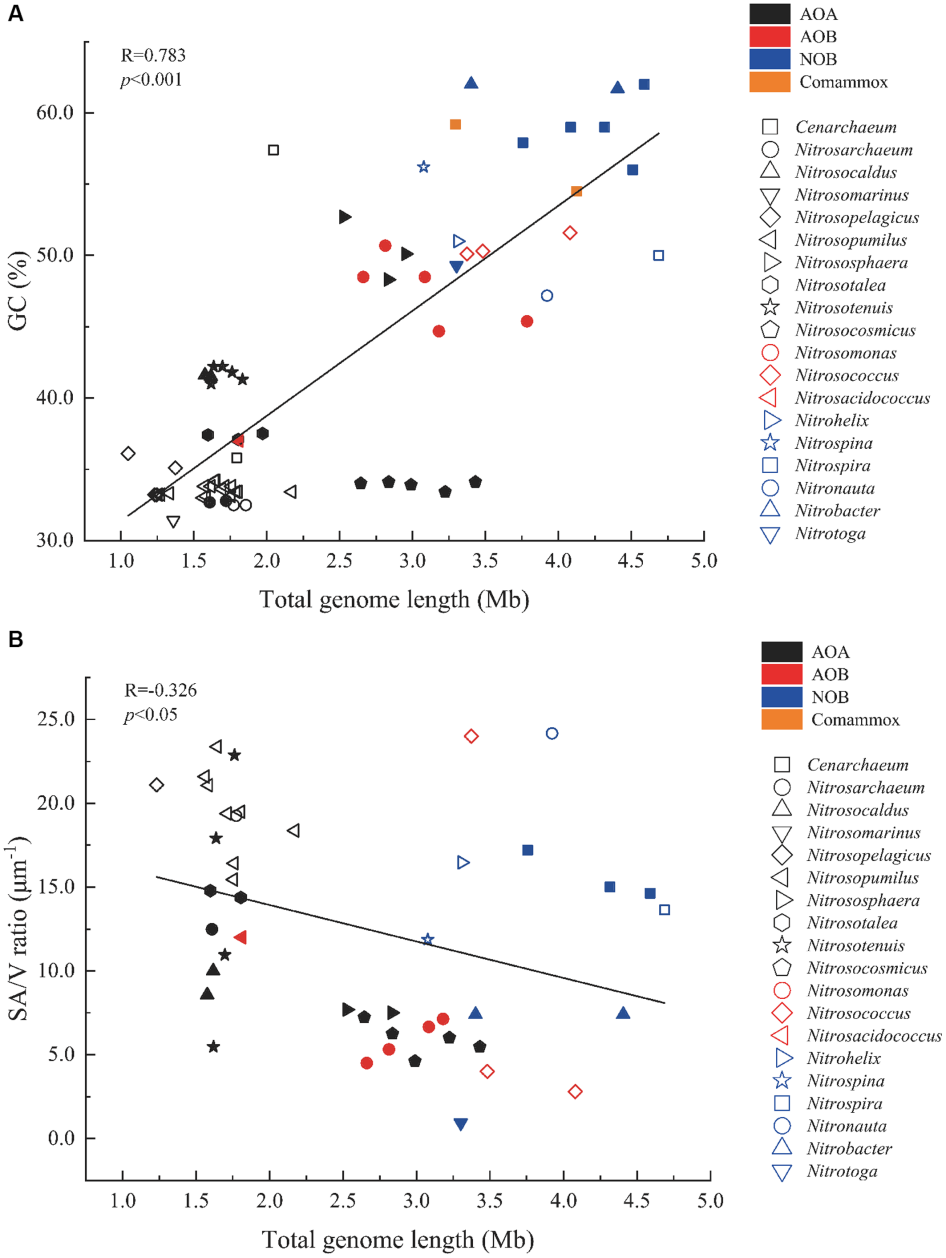
Scatter plots of correlations between **(A)** GC content (%) and total genome length (Mb) and between **(B)** SA/V ratio (μm^−1^) and total genome length (Mb) of selected nitrifiers. The Pearson correlation coefficient (R) of each plot is listed. Filled and open symbols represent strains from terrestrial and marine environments, representatively. AOA, Ammonia-oxidizing archaea; AOB, Ammonia-oxidizing bacteria; NOB, Nitrite-oxidizing bacteria; and Comammox, Complete ammonia oxidizers.

Since functional and genomic characteristics of the comammox were similar to NOB, we grouped them into NOB for the subsequent analysis. The total genome length of AOB (1.81–4.08 Mb, median: 3.16 Mb, *n* = 10) was smaller than NOB (3.08–4.69 Mb, median: 3.91 Mb, *n* = 14; one-way ANOVA and Dunn’s method, *p* < 0.01) but larger than AOA (1.05–3.43 Mb, median: 1.85 Mb, *n* = 46; *p* < 0.01; Figure 2A; Supplementary Table S1). The GC contents of NOB ranging from 47.2 to 62.0% (median: 56.1%, *n* = 14) were greater than AOB ranging from 37.0 to 51.6% (47.1%, *n* = 10; one-way ANOVA and Dunn’s method, *p* < 0.01), and they were both greater than those of AOA (31.4–57.4%, 36.7%, *n* = 46; *p* < 0.01). Only the GC contents of marine AOA *Cenarchaeum symbiosum* A and two terrestrial species *Nitrososphaera viennensis* EN76 and *Ca*. N. evergladensis SR1 were over 50%. Marine AOA exhibited significantly smaller total genome lengths and GC contents than terrestrial ones (*p* < 0.05, Student’s *t*-test), but there was no difference observed between marine and terrestrial AOB or NOB (*p* > 0.05). In addition, it showed that bacterial or archaeal strains of the same genus had similar GC contents and total genome lengths (Figure 2A; Supplementary Table S1). The SA/V ratios of AOA (4.62–23.4 μm^−1^, 13.8 μm^−1^, *n* = 26) were larger than those of AOB (2.79–24.0 μm^−1^, 8.30 μm^−1^, *n* = 8; one-way ANOVA and Dunn’s method, *p* < 0.05), but the ratios of NOB (0.93–24.2 μm^−1^, 12.9 μm^−1^, *n* = 10) were not significantly different from those of AOA and AOB (*p* > 0.05; Figure 2B). The SA/V ratios of collected marine AOA (15.4–23.4 μm^−1^, 19.6 μm^−1^, *n* = 10) were larger than those of terrestrial ones (4.62–22.9 μm^−1^, 10.13 μm^−1^, *n* = 16; one-way ANOVA and Dunn’s method, *p* < 0.01), but the ratios of AOB and NOB did not show the same trend (*p* > 0.05; Figure 2B). The GC content and the SA/V ratio were positively (*R* = 0.783) and negatively (*R* = 0.326) correlated with the genome length, respectively (Pearson’s correlation, *p* < 0.05; Figure 2).

### 3.2 Distributions of *amo* and metabolic genes of representative LDON in genomes of nitrifiers

**Figure 3:**
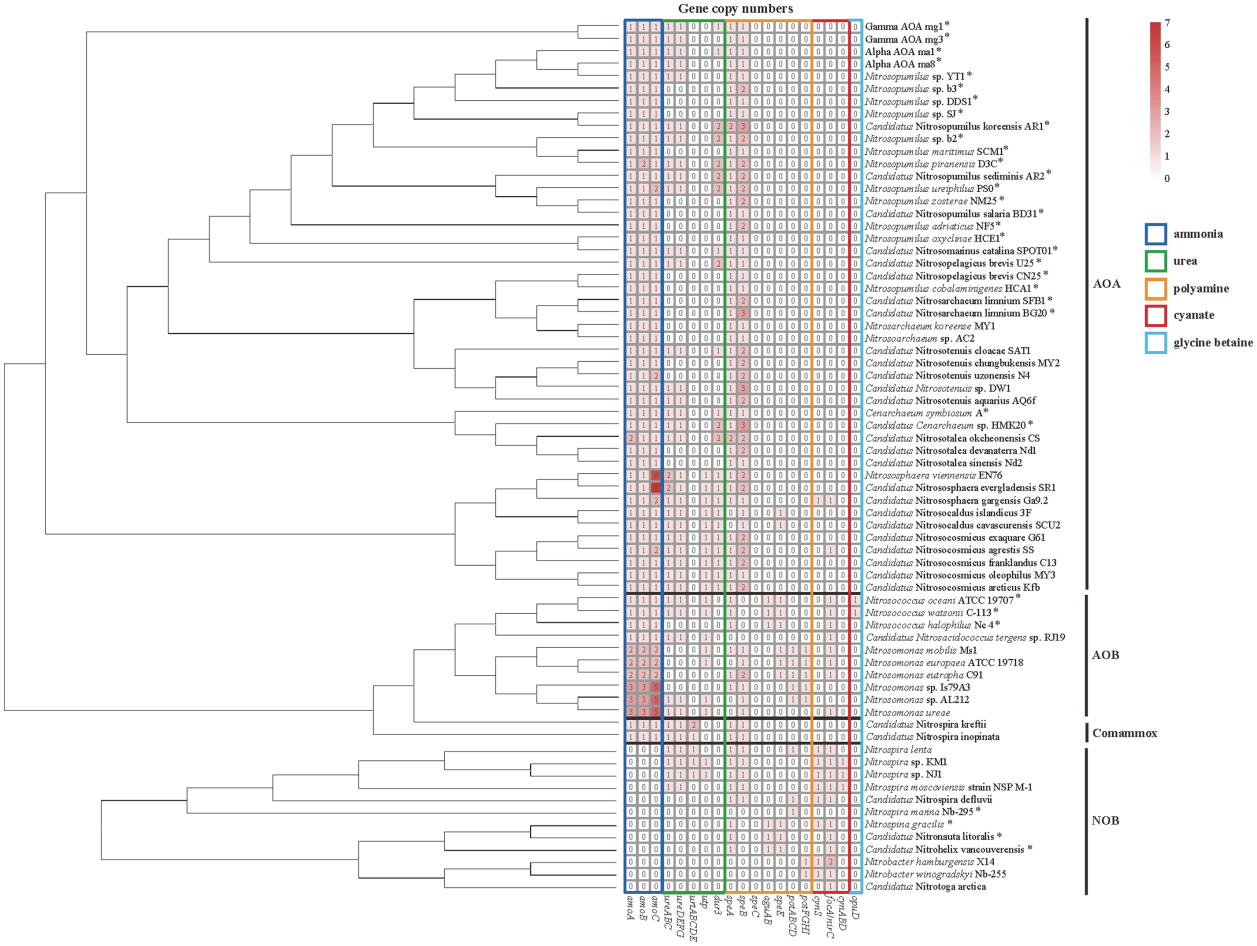
Heatmap of copy numbers of metabolic genes of ammonia (dark blue box), urea (green box), polyamine (orange box), cyanate (red box), and glycine betaine (GBT; light blue box). The strain with an asterisk represents the marine origin. The phylogenetic tree of amino acid sequences of *amoA* is used for the classification of ammonia-oxidizing archaea (AOA), ammonia-oxidizing bacteria (AOB), and complete ammonia oxidizers (Comammox) and that of 16S rRNA gene is used for the classification of nitrite-oxidizing bacteria (NOB).

In AOA, *Ca*. Nitrosotalea okcheonensis CS and *Nitrosopumilus piranensis* D3C had two copies of *amoA* and *amoB*, respectively, and *Nitrosopumilus ureiphilus* PS0, *Ca*. Nitrosotenuis uzonensis N4, *Ca*. Nitrososphaera gargensis Ga9.2, and *Ca*. Nitrosocosmicus agrestis SS contained two copies of *amoC*. Two terrestrial AOA, namely, *Nitrososphaera viennensis* EN76 and *Ca*. N. evergladensis SR1, had six and seven copies of *amoC* gene, respectively (Figure 3). In AOB, all strains in genus *Nitrosococcus* (*n* = 3) and *Ca*. *Nitrosacidococcus tergens* sp. RJ19 only contained one copy of *amoABC*, in contrary to multiple ones in the genus *Nitrosomonas* (Figure 3). Two *Nitrospira* strains of the comammox contained one copy of *amoABC* as well.

**Figure 4:**
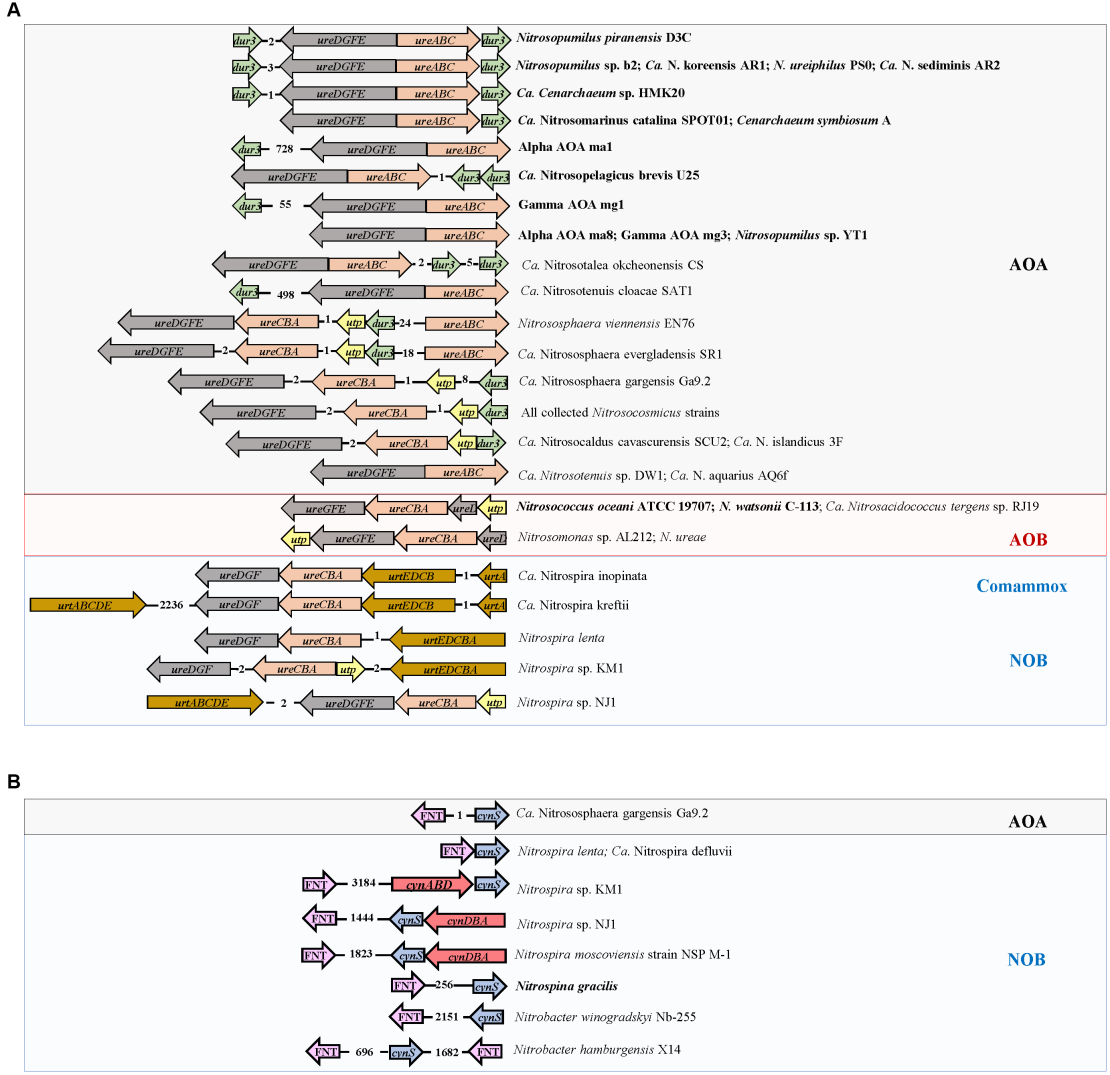
Organization of **(A)** genes encoding urease (*ure*) and urea transporter (*utp*, *dur3*, and *urt*) in genomes of ammonia-oxidizing archaea (AOA) and bacteria (AOB), nitrite-oxidizing bacteria (NOB), and complete ammonia oxidizers (Comammox), and **(B)** genes encoding cyanase (*cynS*), the FNT family (*focA/nirC*), and cyanate ABC transporter (*cynABD*) in genomes of AOA and NOB. The numbers between two genes indicate the number of interval open reading frames (ORFs). The strains highlighted in bold represent marine origin.

In AOA, none of the collected genomes contains bacteria-type urea ABC transporter genes *urtABCDE*. Instead, most harbored the gene *dur3* accompanied by urease-encoding genes *ureABC* and *ureDEFG* (Figure 3). In addition, the genomes of terrestrial genera *Nitrososphaera*, *Nitrosocosmicus*, and *Nitrosocaldus* also contained the gene *utp* (Figure 3). The marine AOA including *Nitrosopumilus* strains which contained the gene *dur3*, *Ca*. Nitrosopelagicus brevis U25 and *Ca*. *Cenarchaeum* sp. HMK 20, and the terrestrial one *Ca*. Nitrosotalea okcheonensis CS had two copies of *dur3*. Two *Nitrososphaera* strains, namely, *Ca*. N. evergladensis SR1 and *N*. *viennensis* EN76, harbored two copies of *ureABC* (Figure 3). The genomes of *Nitrosopumilus* sp. YT1, Alpha AOA ma8, Gamma AOA mg3, *Ca*. *Nitrosotenuis* sp. DW1, and *Ca*. N. aquarius AQ6f harbored urease genes but were not detected with any type of urea transporter genes (Figure 3). All collected strains in the genus *Nitrosarchaeum* did not contain genes related to urea utilization (Figure 3). The ordering of *ureABC* and *ureEFGD* in AOA was contiguous or spaced by a small number of genes (Figure 4A). Except Alpha AOA ma1, Gamma AOA mg1, and *Ca*. Nitrosotenuis cloacae SAT1, the genes *utp* and *dur3* were in close proximity to *ure* in AOA, and *utp* was closer to *ure* than *dur3* when both genes were present (Figure 4A). In AOB, genomes of two *Nitrosococcus* strains, *N*. *oceani* ATCC 19707 and *N*. *watsonii* C-113, four *Nitrosomonas* strains, *N*. *mobilis* Ms1, *N*. *europaea* ATCC 19718, *Nitrosomonas* sp. AL212 and *N*. *ureae*, and *Ca*. *Nitrosacidococcus tergens* sp. RJ19 harbored the gene *utp*, but *N*. *mobilis* Ms1 and *N*. *europaea* ATCC 19718 did not carry *ure* genes (Figure 3). The gene *ureD* was divided from *ureEFG* by *ureABC*, and *utp* was adjacent to *ureD* or *ureG* (Figure 4A). The complete set of urea transporter genes *urtABCDE* was only found in genomes of two comammox, and *Nitrospira* sp. KM1, *Nitrospira* sp. NJ1, and *N*. *lenta* of NOB in the neighbor of *ureABC* and *ureDFG*. The comammox *Ca*. Nitrospira kreftii contained a second copy of *urtABCDE* that was distantly away from *ure* (2236 interval open reading frames; Figures 3, 4A). The gene *utp* was present close to *ureA* in genomes of *Nitrospira* sp. KM1 and *Nitrospira* sp. NJ1 (Figures 3, 4A). The gene *ureE* was absent in genomes of NOB and comammox except in that of *Nitrospira* sp. NJ1 (Figure 4A).

None of the collected AOA and comammox genomes harbored polyamine transporter genes (*potABCD* and *potFGHI*; Figure 3). *Nitrosomonas* strains of AOB contained *potABCD* and *potFGHI* except *N*. *ureae*. *Nitrospira lenta*, *Ca*. N. defluvii, and *N*. *marina* Nb-295 of NOB harbored *potABCD*, while two *Nitrobacter* strains owned *potFGHI* (Figure 3). Genes encoding enzymes for complete pathways of putrescine catabolism were absent in all genomes of collected nitrifiers (Figures 1B, 3). The genomic evidence showed that all AOA, *Nitrosomonas* of AOB, and most *Nitrospira* of NOB and comammox might be capable of synthesizing polyamines intracellularly by arginine decarboxylase [EC: 4.1.1.19] and agmatinase [EC: 3.5.5.11] encoded by *speA* and *speB*, respectively (Figures 1B, 3). Most of AOA contained 2–3 copies of *speB* (Figures 1B, 3). *Nitrosococcus* of AOB, and NOB strains *Nitrospina gracilis*, *Ca*. Nitrohelix vancouverensis, and *Ca*. Nitronauta litoralis were lack of *speB* but owned *aguAB* encoding agmatine deiminase [EC: 3.5.3.12] and N-carbamoylputrescine amidase [EC: 3.5.1.53] for an alternative pathway to synthesize putrescine from agmatine (Figures 1B, 3). The gene *speC* encoding ornithine decarboxylase which could convert ornithine to putrescine was not found in these bacteria and archaea (Figures 1B, 3).

The cyanate ABC-type transporter encoding genes *cynABD* were only detected in *Nitrospira moscoviensis* strain NSP M-1, *Nitrospira* sp. KM1, and *Nitrospira* sp. NJ1 of NOB, accompanied by the gene *cynS* encoding cyanase (Figure 3). The genes *cynABD* were in the neighbor of *cynS* (Figure 4B). In addition, the gene *cynS* was also present in *Ca*. Nitrososphaera gargensis Ga9.2 of AOA, and *Nitrospira lenta*, *Ca*. N. defluvii, *Nitrobacter hamburgensis* X14, and *N*. *winogradskyi* Nb-255 of NOB (Figure 3), but they only contained the FNT family gene *focA*/*nirC*, which were more common in AOB and NOB (Figure 3). Among the nine strains possessing *cynS*, the gene *focA*/*nirC* was adjacent to *cynS in Ca*. N. gargensis Ga9.2, *N*. *lenta*, and *Ca*. N. defluvii (Figure 4B) but was distantly away from *cynS* (over 256 interval open reading frames) in remaining genomes of NOB (Figure 4B).

The GBT transporter gene *opuD* was only detected in genomes of two AOB strains *Nitrosococcus oceani* ATCC 19707 and *N*. *watsonii* C-113 (Figure 3), but genes functioning in GBT synthesis and degradation, such as *betAB*, *gbcAB*, *bhmt*, *grdHI*, and *cdh* (Figure 1E), were absent in all genomes of collected nitrifiers. In additions, genes encoding enzymes involved in the utilization of taurine (*tauACB*, *tauD*, *tpa*, *tauXY*, *xsc*, and *pta*) and methylamine (*tmm*, *mauAB*, *gmaS*, *mgsABC*, and *mgdABCD*; Figures 1D,F) were not found in any genome of collected nitrifiers.

### 3.3 Phylogenetic relationships of key genes involved in urea and cyanate utilization

**Figure 5:**
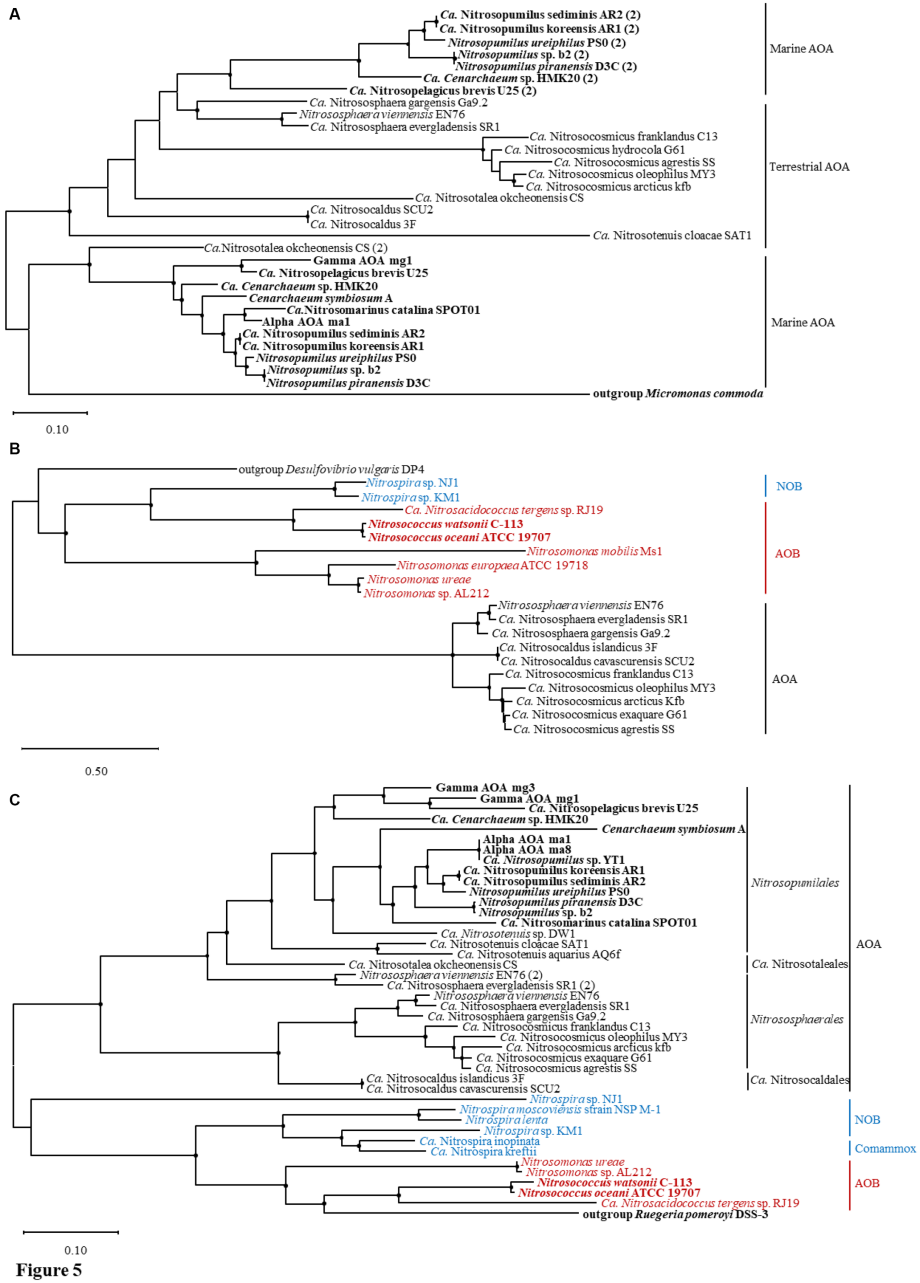
Phylogenetic trees of amino acid fragments of **(A)** *dur3*, **(B)** *utp*, and **(C)** *ureC* identified from ammonia-oxidizing archaea (AOA) and bacteria (AOB), nitrite-oxidizing bacteria (NOB) and complete ammonia oxidizers (Comammox). The trees are constructed with the maximum likelihood method. Bootstrap values based on 1,000 replicates are indicated for the major branches, and the values >50 are shown as black dots. The numbers in brackets indicate the second copy of *dur3* or *ureC* from the same strain. The strains highlighted in bold represent marine origin.

Thirty-one amino acid sequences of *dur3* from AOA strains were analyzed for phylogenetic relationship (Figure 5A). The sequence of *dur3* in the genome of *Micromonas commoda* was selected as the out-group because *dur3* was originally detected in eukaryotic organisms (Coimbra, 2022). In AOA with two copies of *dur3*, we considered the copy closer to *ure* genes as Copy 1 and the other as Copy 2 for subsequential phylogenetic analysis (Figures 4A, 5A). Basically, the sequences from terrestrial and marine AOA were well divided into two clusters except that Copy 2 of *dur3* from marine AOA strains formed a close relationship with those from terrestrial genera *Nitrososphaera* and *Nitrosocaldus* (Figure 5A; Supplementary Table S4). The Copy 1 sequences of *dur3* from marine AOA genus were homogeneous to that of *M*. *commoda*, which also included Copy 2 of *dur3* from a terrestrial strain *Ca*. Nitrosotalea okcheonensis CS (Figure 5A). The phylogenetic analysis grouped 10 amino acid sequences of the gene *utp* from AOAs, 7 from AOBs, and 2 from NOBs. The sequence of *utp* of Deltaproteobacteria *Desulfovibrio vulgaris* DP4 was used as an out-group because it possessed a homologous urea transporter gene (*utp*) found in mammals (Levin et al., 2009; Figure 5B). The sequences of *utp* from NOB and AOB were homologous to that of *D. vulgaris* DP4 and distinguished from the cluster of AOA (Figure 5B). The amino acid sequence of *ureC* from a marine ɑ-proteobacterium *Ruegeria pomeroyi* DSS-3 was used as an out-group for constructing a phylogenetic tree of *ureC* (Figure 5C). *Ruegeria pomeroyi* DSS-3 is a heterotrophic bacterium ubiquitous in marine environments and is capable of degrading urea with urease (Ferrer-González et al., 2023). Similarly, the sequences of *ureC* from archaea and bacteria were distinctively divided into two groups (Figure 5C). In AOA, the sequences from genera *Nitrosocosmicus* and *Nitrososphaera* belonging to the order of *Nitrososphaerales* (Group I.1b) were clustered with *Nitrosocaldus* of the order *Ca*. Nitrosocaldales except for the second copies of *ureC* in genomes of *Nitrososphaera viennensis* EN76 and *Ca*. N. evergladensis SR1. They were more phylogenetically close to sequences from the genus *Nitrosotalea* belonging to the order *Ca*. Nitrosotaleales (Group I.1a-associated) and the order *Nitrosopumilales* mostly comprised of marine AOA (Figure 5C). The amino acid sequences of *ureC* from AOB, NOB, and comammox were tightly clustered and homologous to that of *R. pomeroyi* DSS-3 (Figure 5C).

**Figure 6:**
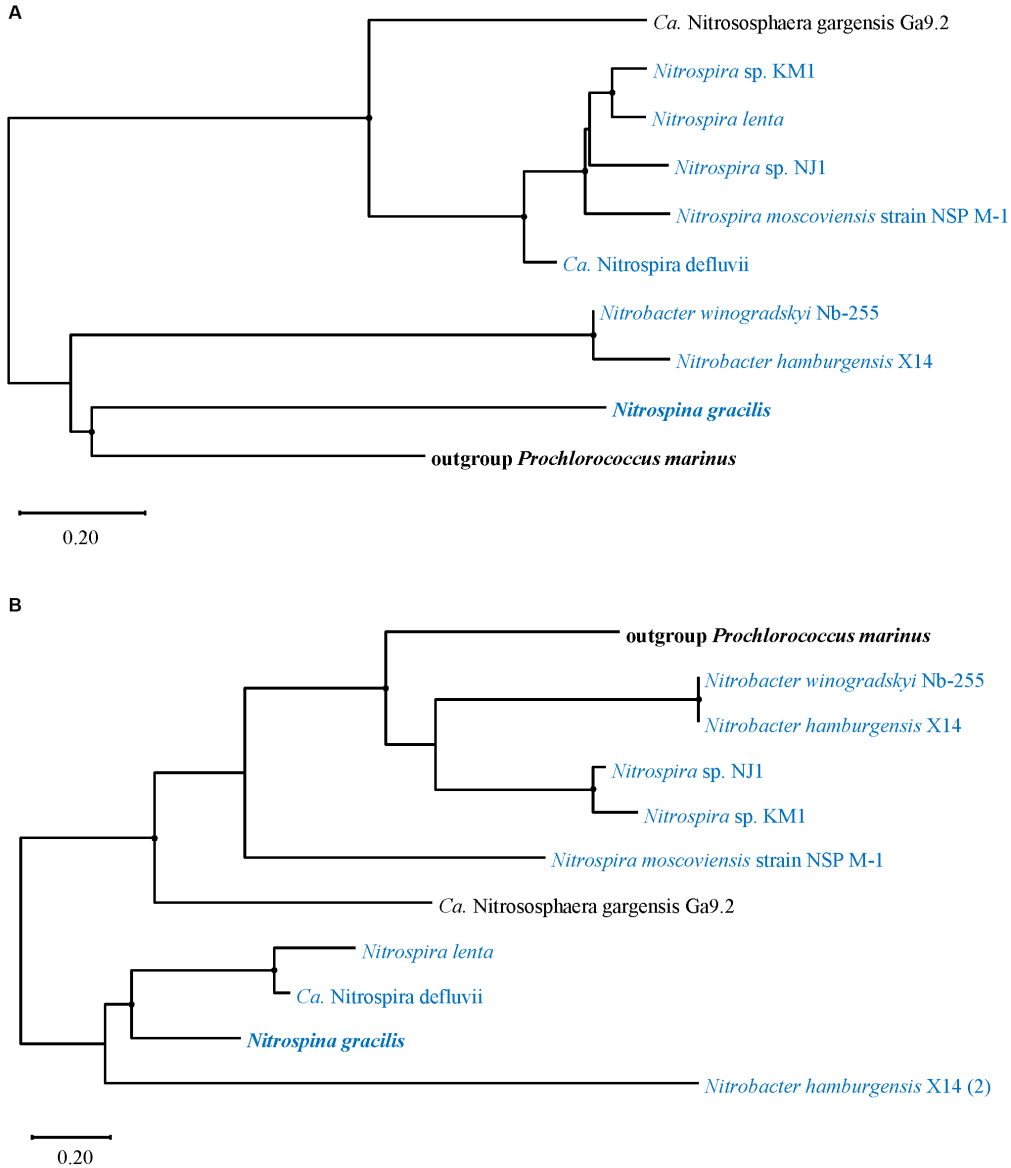
Phylogenetic trees of amino acid fragments of **(A)** *cynS* and **(B)** the FNT family gene *focA/nirC* identified from ammonia-oxidizing archaea (AOA) and nitrite-oxidizing bacteria (NOB). The strains shown in panel **(B)** are selected as ones which also contain *cynS*. The trees are constructed with the maximum likelihood method. Bootstrap values based on 1,000 replicates are indicated for the major branches, and the values >50 are shown as black dots. The numbers in brackets indicate the second copy of *focA/nirC* from the same strain. The strains highlighted in bold represent marine origin.

Amino acid sequences of *cynS* and the FNT family gene *focA*/*nirC* from *Ca*. Nitrososphaera gargensis Ga9.2 and NOB strains were individually analyzed for the phylogenetic relationship with corresponding sequences from the out-group *Prochlorococcus marinus* since the genes were mostly found in marine cyanobacterial strains and their functions had been verified (Maeda and Omata, 2009; Maeda et al., 2015; Figure 6). The *cynS* sequence of *Ca*. N. gargensis Ga9.2 was clustered with those of *Nitrospira* and distinguished from two *Nitrobacter* strains and the marine AOB *Nitrospina gracilis*, which were phylogenetically close to *P*. *marinus* (Figure 6A). Differently, in the phylogenetic tree of the gene *focA*/*nirC*, in addition to three *Nitrospira* strains, *Nitrospira* sp. NJ1, *Nitrospira* sp. KM1, and *N*. *Moscoviensis* strain NSP M-1, the sequence of *Ca*. N. gargensis Ga9.2 was also clustered with those of two Nitrobacter strains, which were closely related to that of *P*. *marinus* (Figure 6B). The gene *focA*/*nirC* of the marine AOB *N*. *gracilis* grouped with those from *Nitrospira lenta* and *Ca*. N. defluvii and the second copy of *focA*/*nirC* in *Nitrobacter hamburgensis* X14 (Figure 6B).

## 4 Discussion

### 4.1 Genomic and phenotypic characteristics of nitrifiers reflecting nitrogen availability and affinity of nitrifiers

Although we did not collect genomes of all nitrifiers, such as those identified by MAG and single-cell sequencing, the genomes of isolated and enriched nitrifier strains as well as several MAGs from extreme environments could act as representatives of typical habitats (Supplementary Table S1). The genomic analysis and comparison were believed to be vigorous to gain insights into differences in physiological and metabolic characteristics among AOA, AOB, NOB, and comammox.

Generally, AOA have smaller genome lengths and lower GC contents than AOB and NOB (Figure 2A; Supplementary Table S1). The environmental differences in ecological niches may lead to this differentiation, in line with survival strategy and nitrogen metabolic capacity (Kitzinger et al., 2020). Previous studies on bacteria showed that those with large chromosomes usually had higher GC contents (Guo et al., 2009). Genomes lacking GC may be beneficial to the survival in nitrogen-limiting environments because AT pairs use one less nitrogen than GC pairs (Luo et al., 2015). Thus, low GC contents in AOA indicated nitrogen limitation in their niches, especially those in marine environments. The limited capacity of using LDON may also prohibit the access of nitrogen to AOA (Figure 3). However, great SA/V ratios of marine AOA suggested a high affinity of ammonia for compensating low concentrations in marine environments. More copies of urea transporter gene *dur3* corresponding to higher SA/V ratios might be a strategy for conquering nitrogen limitation as well (*R* = 0.754, *p* = 0.003, Spearman rank correlation; Supplementary Figure S1). The group *Nitrosocosmicus* had a large genome size (2.64–3.63 Mb) but a low GC content (33.4–34.1%; Figure 2A; Supplementary Table S1) probably due to its extreme low affinity of NH_3_ plus NH_4_^+^ (Jung et al., 2022), although the surrounding environment might not be limited by nitrogen (e.g., waste water plant treatment; Sauder et al., 2017). NOB generally had larger GC contents in response to greater genome lengths (Figure 2A), suggesting either nitrogen sufficiency in their habitats or their great efficiency to absorb substrates from the environment. The larger SA/V ratio of most collected NOB (e.g., *Nitronauta*, *Nitrohelix*, and *Nitrospira*) implied the latter circumstance, but the potential of using diverse nitrogen sources (e.g., urea and cyanate; Figure 3) might enhance their versatility of acquiring nitrogen from the environment, resulting in increased GC content and genome length. The relatively greater GC contents but lower SA/V ratios of AOB compared to AOA could be the consequence of AOB survival in substrate-rich environments with increased nitrogen availability (Soliman and Eldyasti, 2018). Thus, the genomic (e.g., GC%) and phenotypic characteristics (e.g., SA/V) of nitrifiers might reveal their niche partitioning as the basis of their capability and efficiency of nitrogen utilization for environmental adaptation.

### 4.2 Strategy of urea and cyanate utilization being intensely to characteristics of environmental niches and nitrogen demand of nitrifiers

Most of the collected AOA harbor urease genes (*ureABC* and *ureDEFG*; Figure 3); however, urea ABC transporter genes *urtABCDE* commonly detected in bacterial genomes (Veaudor et al., 2019) were not found in any collected AOA. Instead, two types of urea transporter genes *dur3* (Solomon et al., 2010) and *utp* (Bossé et al., 2001) were present in AOA harboring *ure* genes except *Ca*. *Nitrosotenuis* sp. DW1, *Ca*. N. aquarius AQ6f, *Nitrosopumilus* sp. YT1, Alpha AOA ma8, and Gamma AOA mg3 (Figure 3). Since nitrite production has been detected in the enrichment culture of *Ca*. N. aquarius AQ6f with urea (Sauder et al., 2018), it suggests that these five strains may contain unknown urea transporter proteins, or urea could diffuse across the cell membrane without active transports (Sauder et al., 2018). The genes *utp* and *dur3* are both present in genera belonging to Group I.1b (*Nitrososphaera and Nitrosocosmicus*) and *Nitrosocaldus*, while *dur3* is the only urea-transporter gene in genomes of Group I.1a (*Nitrosopumilus*, *Nitrosopelagicus*, and *Nitrosotenuis*) and Group I.1a-associated (*Nitrosotalea*) strains (Figures 3, 4A). The verified growth of two *Nitrososphaera* strains and *Nitrosopumilus ureiphilus* PS0 in media added with urea suggests that the urea transporter encoded by *dur3* is functional; however, whether *dur3* is functional requires further experimental validation because some AOA lacking *dur3* or *utp* can also hydrolyze urea without the known transporters (Sauder et al., 2018). In addition, the presence of two copies of *dur3* in the genus *Nitrosopumilus* except *Nitrosopumilus* sp. YT1, and *Ca*. Nitrosopelagicus brevis U25 from marine environments (Figure 3) could be the result of the low availability of urea, which triggers marine AOA to produce more transporter proteins for efficiently utilizing urea (Offre et al., 2014). The protein Dur3 has been demonstrated to encode a high-affinity urea active transporter in marine unicellular photosynthetic eukaryotes (Solomon et al., 2010). Thus, AOA may be advantageous in urea uptake, corresponding to their higher urea uptake rates in several marine ecosystem (e.g., polar waters, the Gulf of Mexico, and coastal Georgia; Alonso-Sáez et al., 2012; Tolar et al., 2016; Kitzinger et al., 2019). The homogeneity of Copy 2 of *dur3* in marine AOA to those in genomes of terrestrial ones indicates lateral gene transfer (LGT) of genes from terrestrial AOA (Figure 5A). The gene *utp* identified in genomes of some terrestrial AOA appears simultaneously with *dur3* (Figures 3, 4A). Since the protein Utp has been considered as a low-affinity urea transporter (Bossé et al., 2001; Raunser et al., 2009), the presence of *utp* in terrestrial AOA could be due to the complex terrestrial environments (wastewater treatment plant, agricultural soils, mud, etc.) with the detection of high urea concentrations (Rittstieg et al., 2001; Wang et al., 2012); however, whether the urea transporter protein encoded by *utp* functions in AOA as an alternative way of urea transport by Dur3 still needs further experimental verification.

The urea transporter encoding gene *dur3* was not detected in genomes of selected AOB and NOB, but instead it was replaced by *utp* and *urt*, respectively (Figures 3, 4A). Genomes of *Nitrosococcus oceani* ATCC 19707, *N*. *watsonii* C-113, *Nitrosomonas* sp. AL212, *N*. *ureae* and *Ca*. *Nitrosacidococcus tergens* sp. RJ19 of AOB processed both *utp* and *ure*, while terrestrial AOB *Nitrosomonas mobilis* Ms1 and *N*. *europaea* ATCC 19718 only possessed a copy of *utp* (Figure 3). Nitrite has been found rapidly produced from *N*. *oceani* ATCC19707 cultured in the medium with urea replacing ammonia (Koper et al., 2004), suggesting that *utp* functions as a urea transporter. Assimilated urea could be efficiently degraded and used as a source of NH_4_^+^ by AOB. *Nitrosococcus oceani* is an AOB species distributed ubiquitously in the oceanic environment and is an important nitrifier in the OMZ (Lam et al., 2009). It incorporates with anammox bacteria and is responsible for nitrogen loss (Woebken et al., 2008). Thus, *N*. *oceani* may be responsible for urea hydrolyzation and subsequential oxidization of ammonia in the OMZ. Urease activity in *N*. *europaea* ATCC 19718 has been experimentally proven absent (de Boer and Laanbroek, 1989; Sliekers et al., 2004), consistent with the genomic evidence (Figure 3). The presence of *utp* without the co-occurrence of urease genes is probably due to its non-specificity for urea. It may also facilitate the diffusion of urea analogs along their concentration gradients (Levin et al., 2009). The detection of *utp* only in AOB implies the advantage of AOA with both *utp* and *dur3* in utilizing urea in different environments because they may alternate the urea transporter depending on urea concentrations.

Genomes of collected NOB and comammox have a full set of *urt* and *ure* genes (Figures 3, 4A), indicating that they are capable of urea utilization. The ureolytic activity has been observed in the culture of *Nitrospira moscoviensis*, *Ca*. N. nitrosa, and *Ca*. N. nitrificans with urea-containing media (Koch et al., 2015; Van Kessel et al., 2015; Vijayan et al., 2021). The gene cluster *urt* is a high-affinity urea transporter (Valladares et al., 2002), suggesting that *Nitrospira* has a competitive advantage in urea uptake in environments with low urea concentrations. Since *Nitrospira* occur ubiquitously in different terrestrial and aquatic habitats (Latocheski et al., 2022), it is tempting to speculate that reciprocal feeding between *Nitrospira* and AOM could be a common phenomenon in nature, but their contribution to total nitrification in different ecosystems remains to be determined. The marine NOB (*Nitrospira marina* Nb-295, *Ca*. Nitronauta litoralis, *Ca*. Nitrohelix vancouverensis, and *Nitrospina gracilis*) do not contain any urea-related gene (Figure 3); however, although the genome of the type strain *N*. *gracilis* of the genus *Nitrospinae* does not contain ureases genes, other clades of *Nitrospinae* (e.g., *Nitrospinae* Clade 2 in the Gulf of Mexico, Kitzinger et al., 2020) representing the major groups of NOB in marine environments commonly contain complete sets of urea transporter and urease genes, and form an intense relationship of reciprocal feeding with AOM (Koch et al., 2015; Pachiadaki et al., 2017). In addition, in NOB, only *Nitrospira* sp. KM1 and *Nitrospira* sp. NJ1 own *utp* (Figures 3, 4A). The reason that both AOB and NOB lack the gene *dur3* could be due to the fact that *dur3* of AOA was derived from the same ancestor as that of eukaryotes (Figure 5A; Levin et al., 2009; Spang et al., 2012). The gene *utp* of terrestrial AOA may evolve from bacteria through LGT (Figure 5B). Overall, the genes *dur3* and *utp* may be functional and encode proteins for urea transport from extracellular environments; however, as evidence shows that AOM harboring these two genes have a lower urea uptake rate than those with *urt*, such as *Nitrospira* or other heterotrophic bacteria (Vijayan et al., 2021), it suggests that urea may be the alternative energy and nitrogen source of AOM, which still use ammonia or ammonium as their major substrate (Kitzinger et al., 2020). It is noted that most of *Nitrospira* genomes lack *ureE* that serves as a bridge to acquire nickel from hydrogenase maturation factor HypA, which is subsequently donated to UreG. HypA is a metallochaperone and selectively delivers the nickel to the active site (Xiong et al., 2023). The absence of *ureE* may result in low urease activity in NOB (Carter et al., 2009; Fujitani et al., 2020).

The cyanate degradation gene *cynS* was only detected in NOB and one AOA strain *Ca*. Nitrososphaera gargensis Ga9.2 (Figure 3). Although both comammox strains do not contain *cynS* in this study, it has been found that a comammox MAG LK70 owns *cynS* (Yang et al., 2020). The genes *cynABD* encoding a high-affinity cyanate ABC transporter (Maeda and Omata, 2009) were only detected in *Nitrospira* sp. KM1, *Nitrospira* sp. NJ1, and *N*. *moscoviensis* strain NSP M-1 of NOB (Figures 3, 4B), and *N*. *moscoviensis* was proven to use cyanate (Palatinszky et al., 2015). The remaining strains with *cynS* without *cynABD* contained the FNT family encoded by *focA*/*nirC* (Figures 3, 4B), which was hypothesized as the low-affinity cyanate transporter (Rycovska et al., 2012; Spang et al., 2012; Palatinszky et al., 2015). FNT proteins are found in most phyla of bacteria, archaea, and lower eukaryotes (Falke et al., 2010) and are key regulators of the metabolic flow in microorganisms (Wiechert and Beitz, 2017). The formate transporter (FocA) fuels the energy-generating formate hydrogen lyase reaction. Nitrite derived from chemical reduction of nitrate or oxidation of nitrogen monoxide is transported via NirC (Wiechert and Beitz, 2017). Formate/nitrite transporter is also presumed to be permeable for cyanate due to the proximity of genes for transporter and enzymatic degradation (Spang et al., 2012). Thus, the adjacent relationship between *cynS* and *focA*/*nirC* in genomes of *Ca*. Nitrososphaera gargensis Ga9.2, *Nitrospira lenta*, *Ca*. N. defluvii, *Nitrobacter hamburgensis* X14, and *N*. *winogradskyi* Nb-255 (Figure 4B) may suggest the involvement of FNT family gene encoding protein in cyanate transport. However, there is no experimental evidence that those five nitrifiers can use cyanate. The role of FNT family gene as the cyanate transporter is still uncertain with only genomic evidence (Spang et al., 2012). Although it is lack of evidence that the AOM can assimilate and break down cyanate from the environment, a previous study has shown that the pure culture of *Nitrosopumilus maritimus* SCM1 added with ^15^N-cyanate produces ^15^N-ammonium and ^15^N-nitrite, which suggests a process of extracellular breakdown of cyanate by AOM (Kitzinger et al., 2019). It is also reported that NOB supply cyanase-lacking AOM with ammonium from cyanate. The ammonium can be fully nitrified by this microbial consortium through reciprocal feeding in co-culture experiments (Palatinszky et al., 2015). If the FNT family was a cyanate transporter, NOB could be the dominant nitrifier in cyanate utilization and play a key role in reciprocal feeding in nature. Thus, NOB that have high GC contents and SA/V ratios may have more versatility to utilize different extracellular nitrogen to satisfy their nitrogen demands.

### 4.3 Metabolic potentials of other LDON compounds

Polyamines are the primary amines consisting of two or more amine substitutions (Liu et al., 2015; Damashek et al., 2019). They are ubiquitous in cells of all lives and are essential for integral cellular processes, such as nucleic acid synthesis and stabilization, cellular growth, protein synthesis, biofilm formation, and siderophore production (Michael, 2018). They are *de novo* synthesized intracellularly (Figure 1B) and can be directly released from living and dead cells or from protein degradation into environments (Liu et al., 2016; Michael, 2016). Eukaryotic phytoplankton and heterotrophic bacteria (e.g., *Roseobacter* and SAR11) have been detected to utilize extracellular polyamines (Mou et al., 2010; Liu et al., 2016; Noell et al., 2021). Polyamines can be used by bacterioplankton as a nitrogen source (Figure 1B) and contribute to over 4% of bacterial nitrogen demand in aquatic environments (Liu et al., 2015; Krempaska et al., 2018; Madhuri et al., 2019; Liu et al., 2022a). Previous studies observed that putrescine-N could be oxidized and contribute to a significant fraction of total nitrification in coastal waters, and putrescine-N oxidation rate even exceeded that of urea (Damashek et al., 2019). Moreover, the faster polyamine-N oxidation rate than its uptake rate in the same water region as well as the production of ^15^N-NO_2_^−^ in pure cultures of some AOA with ^15^N-putrescine suggest that AOM directly oxidize amine groups of polyamines resembling the pathway of ammonia oxidation (Liu et al., 2015; Damashek et al., 2021) since ammonia monooxygenase can co-metabolize a variety of organic compounds (Rasche et al., 1991; Wright et al., 2020). Thus, it is reasonable that genes homologous to *pot*-encoding polyamine transporters are not detected in selected AOA strains. The protein Dur3 has been found to be capable of transporting polyamines along with urea (Uemura et al., 2007), raising the potential that AOA may also assimilate polyamines; however, since all AOA, *Nitrosomonas* of AOB, and *Nitrospira lenta*, *Ca*. N. defluvii, *N*. *marina* Nb-295, *Nitrobacter hamburgensis* X14, and *N*. *winogradskyi* Nb-255 of NOB, which harbor *pot* genes, lack genes involved in polyamine catabolism (Figures 1B, 3), polyamines may not be degraded into ammonium intracellularly for the following oxidation processes. Instead, assimilated polyamines may serve for other physiological purposes as mentioned above (Kim et al., 2016).

In this study, all selected nitrifiers have the potential to synthesize putrescine either from arginine or agmatine except *Ca*. *Nitrosacidococcus tergens* sp. RJ19 of AOB and *Nitrospira marina* Nb-295, *Nitrobacter hamburgensis* X14, *N*. *winogradskyi* Nb-255, and *Ca*. Nitrotoga arctica of NOB (Figures 1B, 3). Archaea have been mentioned to form branched or long-chain polyamines and induce structural changes to DNA that can facilitate growth in extreme environments (Michael, 2018), or polyamines could be used as a donation of aminobutyl group for the growth of some archaeal halophiles and some methanogens (Michael, 2018). Polyamine synthesis and excretion are significantly up-regulated in AOA grown in environments with high levels of ammonia, which is thought to be one of the reasons for the ammonia tolerance of AOA in terrestrial environments (Liu et al., 2021). AOA may also use polyamines for detoxification (e.g., H_2_O_2_) or form biofilm for substrate uptake (Michael, 2018). More copies of *speB* detected in AOA may be the evolutionary consequence for polyamine synthesis (Magadum et al., 2013). Thus, instead of utilizing polyamine-N as an energy source, AOA might be a significant source of polyamines and contribute to polyamine cycling in different environments.

Taurine dissimilation could be an important source of nitrogen (Denger et al., 2004). *Thaumarchaeota* and *Euryarchaeota* have been reported to assimilate taurine in the upper water column of the northern Adriatic Sea identified by MICRO-CARD-FISH, and the uptake by *Thaumarchaeota* is even beyond that of SAR11 and *Roseobacter* clade in fall when the release of taurine is enhanced by zooplankton (Clifford et al., 2019); however, in this study, none of collected nitrifiers possesses taurine transporter genes *tauACB* and catabolic genes (Figure 1D), suggesting that nitrifiers either assimilate taurine using other transporters (e.g., those for amino acids) or directly break down taurine extracellularly (Damashek et al., 2019, 2021). In this study, only two marine AOB strains *Nitrosococcus oceani* ATCC 19707 and *N*. *watsonii* C-113 own glycine betaine (GBT) transport gene *opuD*. GBT has been found common in bacteria as osmotic molecules (Shakhman and Harries, 2021). A variety of soil and aquatic bacteria have catabolic pathways that convert choline to glycine in multiple steps via GBT (Figure 1E), using both choline and GBT as the sole carbon and nitrogen sources (Wargo, 2013). In addition, bacteria and methanogenic archaea in the cold spring are able to synergistically convert GBT to methane (Li et al., 2021). The lack of GBT transport, synthesis, and degradation genes in most of selected nitrifiers suggests that GBT is not a nitrogen source to nitrifiers but may function in regulating osmotic pressure (Csonka, 1989). Similarly, methylamines which have been considered as important nitrogen and energy sources for heterotrophic bacteria in natural environments (Chen, 2012) may not be utilized by nitrifiers due to the lack of functional genes.

### 4.4 Evolution of LDON metabolism in nitrifiers

It is reported that archaeal ammonia monooxygenases share a more recent evolutionary history with actinobacterial monooxygenases than with those of AOB or comammox (Alves et al., 2018). The *amoA* of the comammox *Nitrospira* was transferred to AOB or that of both bacteria was derived from an unknown third donor (Palomo et al., 2018). The *ureC* gene of these bacteria and archaea probably evolved independently after an early gene duplication event, as did cyanobacteria and eukaryotes (Glass et al., 2009). The acquisition of urease genes may coincide with the gain of ammonia monooxygenase genes during the transition from *Thaumarchaeota* to AOA (Sheridan et al., 2020). Therefore, the potential LGT between bacteria and archaea may not exist in the evolutions of *amoA* and *ureC* (Figures 3, 5C). However, a previous study suggested that a certain amount of thaumarchaeotal gene clusters were recruited from bacteria for overcoming stresses and facilitating the environmental adaptation of *Thaumarchaeota* (Ren et al., 2019). According to the phylogenetic relationships of *utp* (Figure 5B), and two cyanate metabolic genes *cynS* and *focA*/*nirC* (Figure 6) between nitrifiers, it seems that LGT affects these genes and mostly happens between terrestrial AOA and bacteria (Figures 5B, 6). It has been proposed that the UT family is prokaryotic origin, and the encoding gene *utp* in groups of terrestrial AOA (*Nitrososphaerales* and *Ca*. Nitrosocaldales) is probably either transmitted vertically or horizontally acquired from a bacterium (Figure 5B; Minocha et al., 2003). Since the GC content of a newly acquired gene differs from that of the whole genome, a significant difference in the GC content between the gene *utp* (41.6–57.0%, median: 43.5%) and whole genomes of terrestrial AOA (33.4–52.7, 37.8%; *p* = 0.04, Mann–Whitney rank sum test; Supplementary Table S5) indicates a LGT event of *utp* (Lal and Lal, 2010). The adaptation of bacterial *utp* to their respective genomes as depicted by GC contents (*utp*: 35.0–59.0, 48.1%, genome: 37.0–59.0, 50.1%; *p* > 0.05; Supplementary Table S5) suggests that *utp* is bacterial origin and acquired by terrestrial AOA via LGT. GC contents of *cynS* (53.5–59.9, 56.3%) and *focA*/*nirC* (59.4–66.6, 60.3%) in bacteria which contain both genes did not show significant differences from those of whole genomes (56.0–62.0, 59.0%; *p* < 0.05). Similarly, GC contents of the two genes of *Ca*. Nitrososphaera gargensis Ga9.2 follow the same pattern as *Nitrospira* with a relatively lower and higher GC content of *cynS* and *focA/nirC* than that of the whole genome, respectively (Supplementary Table S5). It implies that *Nitrososphaera* and *Nitrospira* may acquire them via an ancient LGT event, which was also supported by good bootstrap values (Figure 6). The significantly greater CAI values of *cynS* (0.65–0.75, 0.66) and *focA/nirC* (0.65–0.76, 0.70) than those of bacterial whole genomes (0.51–0.62, 0.56; *p* < 0.05) further support the idea of LGT and indicate high gene expression in bacteria (Supplementary Table S6). A synchronous acquisition of genes via LGT by *Nitrospira* and terrestrial AOA genera *Nitrsosphaera* could also be verified by *speA* (Supplementary Figure S2) that is involved in the key process of putrescine synthesis (Figure 1B).

It is reported that the emerged AOA progress through an adaptive pathway from terrestrial hot springs to mesophilic soil (∼652 Ma) and then to shallow and deep oceans (∼509 Ma; Yang et al., 2021). The glaciation triggers the evolution of AOA diverging into two groups, one having the mesophilic terrestrial AOA group (genera *Nitrosocosmicus* and *Nitrososphaera*) and the other including marine AOA and acidic soil AOA group (genus *Nitrosotalea*; Yang et al., 2021). The driver for the evolutionary divergence of marine AOA from acidic soil AOA is oxygenation (Yang et al., 2021). The cluster of amino acid sequences of *amoA*, *dur3*, or *ureC* of marine AOA (Figures 3, 5B,C) confirms that marine AOA evolve intimately. However, *dur3* in genomes of AOA, on the other hand, differs from other genes in that the two copies of marine AOA are of different origins according to both phylogenetic relationships and GC contents (Figures 4A, 5A; Supplementary Table S5). It is observed that both copies of *dur3* of marine AOA exhibit higher GC contents (Copy 1: 40.1–43.9, 40.6%; Copy 2: 36.3–40.6, 37.5%) than those of whole genomes (33.2–35.8, 33.6%; calculations only count marine AOA with both copies of *dur3*; *p* < 0.05; Supplementary Table S5), while GC contents of *dur3* of terrestrial AOA (36.3–56.4, 38.9%) are in the same range to those of respective genomes (33.4–52.7, 39.3%; *p* > 0.05). It suggests that *dur3* may originate around the same period of time of *ure* acquisition during the transition from *Thaumarchaeota* to AOA and then diverge into two groups during glaciation events (Yang et al., 2021). The close clustering of Copy 1 of *dur3* of marine AOA and Copy 2 of *Nitrosotalea* verifies this evolutionary process (Figure 5A). Copy 1 of *dur3* of *Nitrosotalea* and Copy 2 of marine AOA are apparently not gained from gene duplication; instead, it could be transferred from terrestrial AOA through LGT. Dur3 orthologues have been detected in higher plants, algae, and fungi (Solomon et al., 2010). The lack of *dur3* in genomes of bacterial nitrifiers, and the clustering of *dur3* sequences of marine AOA and a marine alga *M*. *commoda* (Figure 5A) imply that *dur3* in eukaryotic may evolve from AOA.

## 5 Conclusion

The analysis of diversities and phylogenetic relationships of genes involved in LDON metabolisms in genomes of representative AOA, AOB, NOB, and comammox develops a more holistic understanding of the potentials of LDON metabolism by nitrifiers and sheds light on evolutionary relationships of functional genes involved in these processes. Our data suggest that GC contents, genome sizes, and SA/V ratios of nitrifiers may reflect the availability of nitrogen in their environmental niches and their capability of nitrogen assimilation for environmental adaptation. Our finding reinforces that nitrifiers tend to assimilate and degrade LDON for acquiring nitrogen or reciprocal feeding (e.g., urea and cyanate). They may also directly oxidize amine groups in LDON (e.g., polyamines and taurine) extracellularly to increase their competitive advantage when facing the substrate limitation. They could acquire this capability from early genetic evolution or LGT. Within different groups of nitrifiers, NOB are more advantageous and versatile in nitrogen assimilation than AOM due to their high affinity of ammonia and urea, and potentials in cyanate utilization. They may share similar environmental niches with AOA and form intense reciprocal feeding relationships. In marine environments, AOA could be more efficient in using urea than AOB, which only dominate in environments with high urea concentration. In terrestrial environments, AOA may adjust the protein expression of the urea transporter (Utp or Dur3) to adapt to different urea concentrations, but again AOB only use urea at high concentrations. Our comparative analysis of LDON metabolic genes in different nitrifiers will guide future studies on the isolation and culture of new strains, providing a theoretical basis for their survival strategies in diverse environments. Moreover, it will contribute to model systems to study reciprocal or competitive interactions, which can severely affect matter and energy flows of ecosystems.

## Data availability statement

The original contributions presented in the study are included in the article/Supplementary material, further inquiries can be directed to the corresponding author.

## Author contributions

QL: Conceptualization, Data curation, Funding acquisition, Investigation, Methodology, Project administration, Supervision, Validation, Visualization, Writing – original draft, Writing – review & editing. YC: Data curation, Formal analysis, Investigation, Methodology, Resources, Software, Validation, Visualization, Writing – original draft, Writing – review & editing. X-WX: Project administration, Writing – review & editing.

## Footnotes

^1^ https://www.biologicscorp.com/tools/GCContent/

^2^ http://www.ncbi.nlm.nih.gov

^3^ https://www.biologicscorp.com/tools/CAICalculator
