# Supplementary material for: Genomic insight into strategy, interaction and evolution of nitrifiers in metabolizing key labile-dissolved organic nitrogen in different environmental niches
Source: Front Microbiol. 2023 Dec 13;14:1273211. doi: 10.3389/fmicb.2023.1273211 (PMC10753782; doi:10.3389/fmicb.2023.1273211)
Supplement: Supplementary file 1 [file Table_1.DOCX]

Supplementary Material

**Table S1** Sources, genomic characteristics, culture methods and accession ID of selected ammonia-oxidizing archaea (AOA) and bacteria (AOB), nitrite-oxidizing bacteria (NOB) and complete ammonia oxidizers (Comammox)

| **Strains** | **Sources** | **Genome Size**  **(Mb)** | **GC (%)** | **Number of Coding Sequences** | **Culture method** | **Reference** | **Accession ID** |
| --- | --- | --- | --- | --- | --- | --- | --- |
| **AOA** |  |  |  |  |  |  |  |
| *Nitrosopumilus cobalaminigenes* HCA1 | coastal seawater | 1.56336 | 33.0 | 1956 | Isolate | (Qin et al., 2020) | SAMN08535023 |
| *Nitrosopumilus oxyclinae* HCE1 | coastal seawater | 1.58511 | 33.1 | 1928 | Isolate | (Qin et al., 2020) | SAMN08535025 |
| *Nitrosopumilus ureiphilus* PS0 | marine surface sediment | 2.17142 | 33.4 | 2821 | Isolate | (Qin et al., 2020) | SAMN08535028 |
| *Nitrosopumilus zosterae* NM25 | marine surface sediment | 1.75781 | 33.8 | 2228 | Isolate | (Qin et al., 2020) | SAMD00113975 |
| *Nitrosopumilus maritimus* SCM1 | marine fish tank | 1.64526 | 34.2 | 1986 | Isolate | (Walker et al., 2010) | SAMN03097702 |
| *Nitrosopumilus piranensis* D3C | coastal seawater | 1.71308 | 33.8 | 2230 | Isolate | (Bayer et al., 2019) | SAMN03257648 |
| *Nitrosopumilus adriaticus* NF5 | coastal seawater | 1.80309 | 33.4 | 2295 | Isolate | (Bayer et al., 2019) | SAMN03253153 |
| *Nitrosopumilus* sp. DDS1 | 200-m seawater | 1.75395 | 33.1 | 2782 | Isolate | (Kim et al., 2016) | SAMN03840809 |
| *Candidatus* Nitrosopumilu koreensis AR1 | Arctic seawater | 1.69091 | 33.6 | 2105 | Enrichment | (Park et al., 2014) | SAMN02603137 |
| *Candidatus* Nitrosopumilu sediminis AR2 | Arctic seawater | 1.69091 | 33.6 | 2180 | Enrichment | (Park et al., 2014) | SAMN02603138 |
| *Nitrosopumilus* sp. SJ | 650-m seawater | 1.65083 | 34.2 | 2094 | Enrichment | (Park et al., 2014) | PRJNA66413 |
| *Nitrosopumilus* sp. b2 | beach seawater | 1.61756 | 33.8 | 2081 | Isolate | (Qin et al., 2020) | SAMN08660906 |
| *Nitrosopumilus* sp. b3 | seawater | 1.78907 | 33.4 | 2236 | Enrichment | (Qin et al., 2020) | SAMN08660913 |
| *Candidatus* Nitrosopumilus salaria BD31 | estuary sediment | 1.57296 | 33.8 | 2158 | Enrichment | (Mosier et al., 2012b) | SAMN00016669 |
| *Nitrosopumilus* sp. YT1 (87.7%) | 5000-m seawater | 1.33233 | 33.3 | 1866 | MAG | (Qin et al., 2020) | PRJNA479773 |
| Alpha AOA ma1 (99.1%) | 5080-m seawater | 1.27334 | 33.2 | 1644 | MAG | (Wang et al., 2019) | PRJCA000762 |
| Alpha AOA ma8 (100%) | 10890-m seawater | 1.25613 | 33.2 | 1622 | MAG | (Wang et al., 2019) | PRJCA000762 |
| *Candidatus* Nitrosopelagicus brevis CN25 | seawater | 1.23213 | 33.2 | 1479 | Enrichment | (Santoro et al., 2015) | CP007026 |
| *Candidatus* Nitrosopelagicus brevis U25 | seawater | 1.24554 | 33.2 | 1498 | Enrichment | (Carini et al., 2018) | SAMN04990266 |
| Gamma AOA mg1 (74.9%) | 3000-m seawater | 1.37380 | 35.1 | 1945 | MAG | (Wang et al., 2019) | PRJCA000762 |
| Gamma AOA mg3 (99.8%) | 5900-m seawater | 1.05048 | 36.1 | 1332 | MAG | (Wang et al., 2019) | PRJCA000762 |
| *Candidatus* Nitrosomarinus catalina SPOT01 | seawater | 1.36008 | 31.4 | 1712 | Enrichment | (Ahlgren et al., 2017) | SAMN05730076 |
| *Candidatus Cenarchaeum* sp. HMK20 | seawater | 1.79297 | 35.8 | 2250 | Enrichment | (Qin et al., 2020) | SAMN08660901 |
| *Cenarchaeum symbiosum* A | marine sponge | 2.04509 | 57.4 | 2058 | Enrichment | (Hallam et al., 2006) | DP000238 |
| *Candidatus* Nitrosarchaeum limnium SFB1 | estuary | 1.77272 | 32.5 | 2255 | Enrichment | (Blainey et al., 2011) | CM001158.1 |
| *Candidatus* Nitrosarchaeum limnium BG20 | estuary sediment | 1.85556 | 32.5 | 2642 | Enrichment | (Mosier et al., 2012a) | AHJG00000000 |
| *Nitrosarchaeum koreense* MY1 | soil | 1.60770 | 32.7 | 1975 | Enrichment | (Kim et al., 2011) | SAMN02470178 |
| *Nitrosoarchaeum* sp. AC2 | lake sediment | 1.72073 | 32.8 | 2201 | Enrichment | (Qin et al., 2020) | SAMN09531786 |
| *Candidatus* Nitrosotenuis cloacae SAT1 | wastewater | 1.62016 | 41.0 | 1908 | Enrichment | (Li et al., 2016) | SAMN03286947 |
| *Candidatus* Nitrosotenuis chungbukensis MY2 | soil | 1.76297 | 41.8 | 2124 | Isolate | (Jung et al., 2014) | SAMN02767256 |
| *Candidatus Nitrosotenuis* sp. DW1 | lake sediment | 1.83484 | 41.3 | 2220 | Enrichment | (Qin et al., 2020) | SAMN09531785 |
| *Candidatus* Nitrosotenuis uzonensis N4 | hot spring | 1.63613 | 42.2 | 1970 | Enrichment | (Lebedeva et al., 2013) | CBTY000000000.1 |
| *Candidatus* Nitrosotenuis aquarius AQ6f | freshwater aquarium | 1.69721 | 42.2 | 2043 | Enrichment | (Sauder et al., 2018) | CP024808.1 |
| *Candidatus* Nitrosotalea devanaterra Nd1 | agricultural soil | 1.80530 | 37.1 | 2133 | Isolate | (Herbold et al., 2017) | LN890280 |
| *Candidatus* Nitrosotalea sinensis Nd2 | acidic soil | 1.59805 | 37.4 | 1933 | Isolate | (Herbold et al., 2017) | ERS1465381 |
| *Candidatus* Nitrosotalea okcheonensis CS | acidic soil | 1.97259 | 37.5 | 2471 | Enrichment | (Herbold et al., 2017) | SAMEA20449168 |
| *Nitrososphaera viennensis* EN76 | soil | 2.52794 | 52.7 | 3140 | Isolate | (Kerou et al., 2016) | SAMN02721150 |
| *Candidatus* Nitrososphaera gargensis Ga9.2 | hot spring | 2.83387 | 48.3 | 4056 | Isolate | (Spang et al., 2012) | SAMN02603264 |
| *Candidatus* Nitrososphaera evergladensis SR1 | agricultural area | 2.95437 | 50.1 | 3777 | Enrichment | (Zhalnina et al., 2014) | SAMN03081530 |
| *Candidatus* Nitrososphaera oleophilus MY3 | coal-contaminated sediment | 3.43071 | 34.1 | 4063 | Isolate | (Jung et al., 2016) | SAMN03074222 |
| *Candidatus* Nitrosocosmicus exaquare G61 | wastewater | 2.98978 | 33.9 | 3434 | Enrichment | (Sauder et al., 2017) | SAMN04606696 |
| *Candidatus* Nitrosocosmicus franklandus C13 | agricultural soil | 2.83645 | 34.1 | 3282 | Enrichment | (Nicol et al., 2019) | SAMN11312109 |
| *Candidatus*Nitrosocosmicus agrestis SS | mineral salts | 3.22450 | 33.4 | 4106 | Enrichment | (Liu et al., 2019) | VUYS00000000 |
| *Candidatus* Nitrosocosmicus arcticus Kfb | arctic soil | 2.64486 | 34.0 | 3222 | Enrichment | (Alves et al., 2019) | PRJNA505990 |
| *Candidatus* Nitrosocaldus cavascurensis SCU2 | hot spring | 1.57728 | 41.6 | 1782 | Enrichment | (Abby et al., 2018) | SAMEA104466896 |
| *Candidatus* Nitrosocaldus islandicus 3F | hot spring | 1.61739 | 41.5 | 1814 | Enrichment | (Daebeler et al., 2018) | SAMEA104550428 |
| **AOB** |  |  |  |  |  |  |  |
| *Nitrosococcus oceani* ATCC 19707 | Atlantic Ocean | 3.48169 | 50.3 | 3589 | Enrichment | (Klotz et al., 2006) | SAMN02598329 |
| *Nitrosococcus watsonii* C-113 | bay sediment | 3.37329 | 50.1 | 3432 | Enrichment | (Campbell et al., 2011) | PRJNA40331 |
| *Nitrosococcus halophilus* Nc 4 | bay sediment | 4.07943 | 51.6 | 4228 | Enrichment | (Campbell et al., 2011) | PRJNA36589 |
| *Nitrosomonas mobilis* Ms1 | brackish water | 3.08286 | 48.5 | 3330 | Isolate | (Thandar et al., 2016) | SAMEA4505502 |
| *Nitrosomonas europaea* ATCC 19718 | salt | 2.81209 | 50.7 | 2912 | Isolate | (Chain et al., 2003) | PRJNA52 |
| *Nitrosomonas eutropha* C91 | sewage disposal | 2.66106 | 48.5 | 2884 | Isolate | (Stein et al., 2007) | PRJNA13913 |
| *Nitrosomonas* sp. Is79A3 | freshwater sediment | 3.78344 | 45.4 | 3901 | Isolate | (Bollmann et al., 2013) | PRJNA52837 |
| *Nitrosomonas* sp. AL212 | activated-sludge | 3.18053 | 44.7 | 3417 | Isolate | (Yuichi et al., 2011) | PRJNA32989 |
| *Nitrosomonas ureae* | soil | 3.30782 | 44.5 | 3419 | Isolate | (Kozlowski et al., 2016) | PRJNA323344 |
| *Candidatus Nitrosacidococcus tergens* sp. RJ19 | biofilter unit | 1.81137 | 37.0 | 1729 | Enrichment | (Picone et al., 2021) | PRJEB36691 |
| **Comammox** |  |  |  |  |  |  |  |
| *Candidatus* Nitrospira inopinata | pipe wall | 3.29512 | 59.2 | 3367 | Enrichment | (Daims et al., 2015) | PRJEB10818 |
| *Candidatus* Nitrospira kreftii | aquaculture system biofilter | 4.12573 | 54.5 | 4457 | Enrichment | (Sakoula et al., 2021) | PRJNA575653 |
| **NOB** |  |  |  |  |  |  |  |
| *Nitrospira lenta* | wastewater | 3.75619 | 57.9 | 3725 | Isolate | (Sakoula et al., 2018) | PRJEB26290 |
| *Candidatus* Nitrospira defluvii | wastewater | 4.31708 | 59.0 | 4309 | Enrichment | (Lücker et al., 2010) | FP929003 |
| *Nitrospira moscoviensis* strain NSP M-1 | corroded area of an iron pipe | 4.58949 | 62.0 | 4669 | Isolate | (Koch et al., 2015) | CP011801 |
| *Nitrospira* sp. KM1 | fixed-bed column | 4.50922 | 56.0 | 4787 | Isolate | (Fujitani et al., 2020) | PRJDB5488 |
| *Nitrospira* sp. NJ1 | activated sludge | 4.08482 | 59.0 | 4026 | Isolate | (Ushiki et al., 2018) | LT828648 |
| *Nitrospira marina* Nb-295 | 206-m  seawater | 4.68921 | 50.0 | 5016 | Isolate | (Bayer et al., 2021) | PRJNA262287 |
| *Nitrospina gracilis* | seawater | 3.07771 | 56.2 | 3133 | Isolate | (Lücker et al., 2013) | PRJEB1269 |
| *Candidatus* Nitrohelix vancouverensis | coastal sediment | 3.31007 | 51.0 | 3216 | Isolate | (Mueller et al., 2021) | PRJNA602816 |
| *Candidatus* Nitronauta litoralis | coastal sediment | 3.92164 | 47.2 | 3589 | Isolate | (Mueller et al., 2021) | SAMN13976151 |
| *Nitrobacter hamburgensis* X14 | soil | 4.40697 | 61.7 | 4774 | Isolate | (Starkenburg et al., 2008) | CP000319 |
| *Nitrobacter winogradskyi* Nb-255 | soil | 3.40209 | 62.0 | 3627 | Isolate | (Starkenburg et al., 2006) | PRJNA13474 |
| *Candidatus* Nitrotoga arctica | soil | 3.30105 | 49.3 | 3459 | Enrichment | (Keuter et al., 2022) | SAMEA10456467 |

**Table S2** Cell shapes, lengths, widths and volumes of selected ammonia-oxidizing archaea (AOA) and bacteria (AOB), nitrite-oxidizing bacteria (NOB) and complete ammonia oxidizers (Comammox). The stains with no cell volume reported are due to the lack of the description of cell characteristics in literatures. The strain with an asterisk represents the marine origin.

| **Strain name** | **Shape** | **Cell length and width**  **(µm)** | **Cell volume**  **(****µm^3^)** | **Reference** |
| --- | --- | --- | --- | --- |
| **AOA** |  |  |  |  |
| *Nitrosopumilus cobalaminigenes* HCA1* | rod | length: 0.65 - 1.27  width: 0.15 - 0.26 | 0.0317 | (Qin et al., 2017) |
| *Nitrosopumilus oxyclinae* HCE1* | rod | length: 0.69 - 0.93  width: 0.17 - 0.26 | 0.0294 | (Qin et al., 2017) |
| *Nitrosopumilus ureiphilus* PS0* | rod | length: 0.76 - 1.59  width: 0.22 - 0.26 | 0.0531 | (Qin et al., 2017) |
| *Nitrosopumilus zosterae* NM25* | rod | length: 0.40 - 0.90  width: 0.20 - 0.40 | 0.0459 | (Matsutani et al., 2011) |
| *Nitrosopumilus maritimus* SCM1* | rod | length: 0.50 - 0.90  width: 0.17 - 0.22 | 0.0209 | (Könneke et al., 2005) |
| *Nitrosopumilus piranensis* D3C* | rod | length: 0.49 - 2.00  width: 0.20 - 0.25 | 0.0495 | (Bayer et al., 2019) |
| *Nitrosopumilus adriaticus* NF5* | rod | length: 0.59 - 1.74  width: 0.20 - 0.25 | 0.0463 | (Bayer et al., 2019) |
| *Nitrosopumilus* sp. DDS1* | rod | length: 0.10 - 1.80  width: 0.20 - 0.40 | 0.0671 | (Kim et al., 2016) |
| *Candidatus* Nitrosopumilu koreensis AR1* | - | - | - | - |
| *Candidatus* Nitrosopumilu sediminis AR2* | - | - | - | - |
| *Nitrosopumilus* sp. SJ * | - | - | - | - |
| *Nitrosopumilus* sp. b2* | - | - | - | - |
| *Nitrosopumilus* sp. b3* | - | - | - | - |
| *Nitrosopumilus* sp. b1* | - | - | - | - |
| *Candidatus* Nitrosopumilus salaria BD31* | - | - | - | - |
| *Nitrosopumilus* sp. YT1* | - | - | - | - |
| Alpha AOA ma1* | - | - | - | - |
| Alpha AOA ma8* | - | - | - | - |
| *Candidatus* Nitrosopelagicus brevis CN25* | rod | length: 0.60 - 1.00  width: 0.17 - 0.26 | 0.0290 | (Santoro et al., 2015) |
| *Candidatus* Nitrosopelagicus brevis U25* | - | - | - | - |
| Gamma AOA mg1* | - | - | - | - |
| Gamma AOA mg3* | - | - | - | - |
| *Candidatus* Nitrosomarinus catalina SPOT01* | - | - | - | - |
| *Cenarchaeum symbiosum* A* | - | - | - | - |
| *Candidatus* Nitrosarchaeum limnium SFB1* | rod | length: 0.77  width: 0.24 | 0.0348 | (Mosier et al., 2012c) |
| *Candidatus* Nitrosarchaeum limnium BG20* | - | - | - | - |
| *Nitrosarchaeum koreense* MY1 | rod | length: 0.60 - 1.00  width: 0.30 - 0.50 | 0.101 | (Jung et al., 2011) |
| *Nitrosoarchaeum* sp. AC2 | - | - | - | - |
| *Candidatus* Nitrosotenuis cloacae SAT1 | spherical | length: 1.10 ± 0.10  width: 1.10 ± 0.10 | 0.697 | (Li et al., 2016) |
| *Candidatus* Nitrosotenuis chungbukensis MY2 | rod | length: 0.70  width: 0.20 | 0.0220 | (Jung et al., 2014) |
| *Candidatus Nitrosotenuis* sp. DW1 | - | - | - | - |
| *Candidatus* Nitrosotenuis uzonensis N4 | rod | length: 0.40 - 1.70  width: 0.20 - 0.30 | 0.0515 | (Lebedeva et al., 2013) |
| *Candidatus* Nitrosotenuis aquarius AQ6f | rod | length: 0.60 - 3.60  width: 0.40 | 0.264 | (Sauder et al., 2018) |
| *Candidatus* Nitrosotalea devanaterra Nd1 | rod | length: 0.89 ± 0.05  width: 0.33 ± 0.01 | 0.0761 | (Lehtovirta-Morley et al., 2011) |
| *Candidatus* Nitrosotalea sinensis Nd2 | rod | length: 0.50 - 1.00  width: 0.33 ± 0.01 | 0.0641 | (Lehtovirta-Morley et al., 2014) |
| *Candidatus* Nitrosotalea okcheonensis CS | - | - | - | - |
| *Nitrososphaera viennensis* EN76 | spherical | length: 0.78 ± 0.13  width: 0.78 ± 0.13 | 0.248 | (Stieglmeier et al., 2014) |
| *Candidatus* Nitrososphaera gargensis Ga9.2 | spherical | length: 0.40 - 1.20  width: 0.40 - 1.20 | 0.268 | (Kerou et al., 2016) |
| *Candidatus* Nitrososphaera evergladensis SR1 | - | - | - | - |
| *Candidatus* Nitrososphaera oleophilus MY3 | spherical | length: 1.10  width: 1.10 | 0.697 | (Jung et al., 2016) |
| *Candidatus* Nitrosocosmicus exaquare G61 | spherical | length: 1.30  width: 1.30 | 1.15 | (Sauder et al., 2017) |
| *Candidatus* Nitrosocosmicus franklandus C13 | spherical | length: 0.96  width: 0.96 | 0.463 | (Lehtovirta-Morley et al., 2016) |
| *Candidatus*Nitrosocosmicus agrestis SS | spherical | length: 0.96  width: 0.96 | 0.523 | (Liu et al., 2019) |
| *Candidatus* Nitrosocosmicus arcticus Kfb | spherical | length: 0.83  width: 0.83 | 0.299 | (Alves et al., 2019) |
| *Candidatus* Nitrosocaldus cavascurensis SCU2 | spherical | length: 0.60 - 0.80  width: 0.60 - 0.80 | 0.180 | (Abby et al., 2018) |
| *Candidatus* Nitrosocaldus islandicus 3F | spherical | length: 0.50 - 0.70  width: 0.50 - 0.70 | 0.113 | (Daebeler et al., 2018) |
| **AOB** |  |  |  |  |
| *Nitrosococcus oceani* ATCC 19707* | spherical | length: 1.50  width: 1.50 | 1.77 | (Klotz et al., 2006) |
| *Nitrosococcus watsonii* C-113* | spherical | length: 1.80 - 2.20  width: 1.80 - 2.20 | 0.523 | (Watson, 1965) |
| *Nitrosococcus halophilus* Nc 4* | spherical | length: 1.80 - 2.50  width: 1.80 - 2.50 | 5.20 | (Koops et al., 1990) |
| *Nitrosomonas mobilis* Ms1 | spherical | length: 0.80 - 1.00  width: 0.80 - 1.00 | 0.382 | (Fujitani et al., 2015) |
| *Nitrosomonas europaea* ATCC 19718 | rod | length: 1.50  width: 1.00 | 1.18 | (Lewis and Pramer, 1958) |
| *Nitrosomonas eutropha* C91 | rod | length: 1.60 - 2.30  width: 1.00 - 1.30 | 2.02 | (Stein et al., 2007) |
| *Nitrosomonas* sp. Is79A3 | - | - | - | - |
| *Nitrosomonas* sp. AL212 | rod | length: 1.40  width: 0.70 | 0.539 | (Suwa et al., 1997) |
| *Nitrosomonas ureae* | - | - | - | - |
| *Candidatus Nitrosacidococcus tergens* sp. RJ19 | spherical | length: 0.5  width: 0.5 | 0.0654 | (Picone et al., 2021) |
| **Comammox** |  |  |  |  |
| *Candidatus* Nitrospira inopinata | - | - | - | - |
| *Candidatus* Nitrospira kreftii | - | - | - | - |
| **NOB** |  |  |  |  |
| *Nitrospira lenta* | rod | length: 1.00 - 2.30  width: 0.20 - 0.30 | 0.0810 | (Sakoula et al., 2018) |
| *Candidatus* Nitrospira defluvii | rod | length: 0.70 - 1.70  width: 0.20 - 0.40 | 0.0848 | (Spieck et al., 2006) |
| *Nitrospira moscoviensis* strain NSP M-1 | rod | length: 0.9 - 2.2  width: 0.2 - 0.4 | 0.110 | (Ehrich et al., 1995) |
| *Nitrospira* sp. KM1 | - | - | - | - |
| *Nitrospira* sp. NJ1 | - | - | - | - |
| *Nitrospira marina* Nb-295* | rod | length: 0.8 - 1.0  width: 0.3 - 0.4 | 0.0865 | (Spieck et al., 2006) |
| *Nitrospina gracilis** | rod | length: 2.70 - 6.50  width: 0.30 - 0.40 | 0.442 | (Watson and Waterbury, 1971) |
| *Candidatus* Nitrohelix vancouverensis* | rod | length: 2.70 - 6.50  width: 0.30 - 0.40 | 0.212 | (Mueller et al., 2021) |
| *Candidatus* Nitronauta litoralis* | rod | length: 1.5  width: 0.15-0.2 | 0.0361 | (Mueller et al., 2021) |
| *Nitrobacter hamburgensis* X14 | rod | length: 1.2 - 2.0  width: 0.5 - 0.8 | 0.531 | (Bock et al., 1983) |
| *Nitrobacter winogradskyi* Nb-255 | rod | length: 1.2 - 2.0  width: 0.5 - 0.8 | 0.531 | (Bock et al., 1983) |
| *Candidatus* Nitrotoga arctica | rod | length: 1.0  width: 0.4–0.7 | 2.375 | (Alawi et al., 2007) |

**Table S3** Sources of reference sequences of target genes used in Blastp. The bacterial reference sequences are used for Blastp when no archaeal reference sequence is available. The number in parentheses is the NCBI taxonomy ID.

| **Compounds** | **Genes** | **Reference genomes of archaea** | **Reference genomes of bacteria** |
| --- | --- | --- | --- |
| Ammonia | *amoABC* | *Nitrosopumilus maritimus* SCM1  (NCBI:txid436308) | *Nitrosococcus oceani* ATCC 19707  (NCBI:txid323261) |
| Urea | *ureABC, ureDEFG* | *Nitrosopumilus ureiphilus* PS0  (NCBI:txid1470067) | *Nitrosococcus oceani* ATCC 19707  (NCBI:txid323261) |
|  | *urtABCDE* | *Haloarcula marismortui* ATCC 43049  (NCBI:txid272569) | *Nitrospira lenta*  (NCBI:txid1436998) |
|  | *utp* | *Nitrososphaera viennensis* EN76  (NCBI:txid926571) | *Nitrosococcus oceani* ATCC 19707  (NCBI:txid323261) |
|  | *dur3* | *Cenarchaeum symbiosum* A  (NCBI:txid414004) | *Saccharomyces cerevisiae*  (NCBI:txid4932) |
| Polyamine | *speA, speB* | *Nitrosopumilus maritimus* SCM1  (NCBI:txid436308) | *Nitrosomonas mobilis* Ms1  (NCBI:txid51642) |
|  | *speC* | - | *Candidatus* Pelagibacter ubique HTCC1062  (NCBI:txid335992) |
|  | *speE* | *Candidatus* Nitrosocaldus cavascurensis SCU2  (NCBI:txid2058097) | *Nitrosomonas mobilis* Ms1  (NCBI:txid51642) |
|  | *speG* | - | *Escherichia coli*  (NCBI:txid562) |
|  | *aguA* | *Methanogenium* sp. MK-MG  (NCBI:txid2599926) | *Nitrospirales bacterium*  (NCBI:txid2358460) |
|  | *aguB* | *Euryarchaeota archaeon*  (NCBI:txid2026739) | *Nitrosococcus oceani* ATCC 19707  (NCBI:txid323261) |
|  | *potABCD* | *Halodesulfurarchaeum formicicum*  (NCBI:txid1873524) | *Nitrosomonas eutropha* C91  (NCBI:txid335283) |
|  | *potFGHI* | - | *Nitrosomonas eutropha* C91  (NCBI:txid335283) |
|  | *puuA* | - | *Martelella mediterranea* DSM 17316  (NCBI:txid1122214) |
|  | *spuC* | - | *Rhodobacteraceae bacterium* HLUCCA09  (NCBI:txid1666915) |
|  | *kauB* | - | *Aeromonas hydrophila*  (NCBI:txid644) |
|  | *gabT* | *Nitrosopumilus maritimus* SCM1  (NCBI:txid436308) | *Candidatus* Pelagibacter ubique HTCC1062  (NCBI:txid335992) |
|  | *spdH* | - | *Pseudomonas aeruginosa*  (NCBI:txid287) |
| Cyanate | *cynS* | *Candidatus* Nitrososphaera gargensis Ga9.2  (NCBI:txid1237085) | *Escherichia coli*  (NCBI:txid562) |
|  | *cynABD* | - | *Cyanobacterium* sp. HL-69  (NCBI:txid2054282) |
|  | FNT family gene (*focA*/*nirC*) | *Candidatus* Nitrososphaera gargensis Ga9.2  (NCBI:txid1237085) | *Nitrospira lenta*  (NCBI:txid1436998) |
| Taurine | *tauABC* | *Methanosarcinales archaeon*  (NCBI:txid2250255) | *Candidatus* Pelagibacter ubique HTCC1062  (NCBI:txid335992) |
|  | *tauD* | *Halalkaliarchaeum desulfuricum*  (NCBI:txid2055893) | *Nitrospira defluvii*  (NCBI:txid330214) |
|  | *tpa* | *Candidatus* Thorarchaeota archaeon AB_25  (NCBI:txid1837170) | *Roseobacter* sp. GAI101  (NCBI:txid391589) |
|  | *xsc* | *Nitrosopumilaceae archaeon*  (NCBI:txid2202732) | *Roseobacter cerasinus*  (NCBI:txid2602289) |
|  | *pta* | *Thaumarchaeota archaeon*  (NCBI:txid2026795) | *Nitrospiraceae bacterium*  (NCBI:txid2026770) |
|  | *tauX*, *tauY* | - | *Paracoccus denitrificans*  (NCBI:txid266) |
| Glycine betaine | *betA* | *Methanosphaerula palustris* E1-9c  (NCBI:txid521011) | *Candidatus* Pelagibacter giovannonii  (NCBI:txid2563896) |
|  | *betB* | *Methanoregula boonei* 6A8  (NCBI:txid456442) | *Candidatus* Pelagibacter ubique HTCC1062  (NCBI:txid335992) |
|  | *gbcAB* | - | *Pseudomonas aeruginosa* PA96  (NCBI:txid1457392) |
|  | *bhmt* | - | *Pseudomonas* sp. GX19020  (NCBI:txid2942277) |
|  | *grdHI* | - | *Oribacterium* sp. oral taxon 078 str. F0262  (NCBI:txid608534) |
|  | *cdh* | - | *Pseudomonas aeruginosa* PAO1  (NCBI:txid208964) |
|  | *opuD* | *Halobacterium salinarum* NRC-1  (NCBI:txid64091) | *Nitrosococcus oceani* ATCC 19707  (NCBI:txid323261) |
| Methylamines | *tmm* | - | *Roseobacter denitrificans OCh 114*  (NCBI:txid375451) |
|  | *gmaS* | - | *Rubellimicrobium thermophilum DSM 16684*  (NCBI:txid1123069) |
|  | *mgsA* | - | *Methylophilaceae bacterium*  (NCBI:txid2030816) |
|  | *mgsB* | - | *Donghicola eburneus*  (NCBI:txid393278) |
|  | *mgsC* | *Methanosarcinales archaeon*  (NCBI:txid2250255) | *Burkholderiales bacterium*  (NCBI:txid1891238) |
|  | *mgdABCD* | - | *Rhizobiaceae bacterium*  (NCBI:txid1913961) |
|  | *mauA* | - | *Methylobacillus flagellates*  (NCBI:txid405) |
|  | *mauB* | - | *Methylorubrum extorquens CM4*  (NCBI:txid440085) |

**Table S4** Identity percentages of *dur3* in genomes of ammonia-oxidizing archaea (AOA). Reference sequences (Ref) 1 and 2 are first copy and the second copy of the gene *dur3* in reference genome of *Nitrosopumilus ureiphilus* PS0, respectively. Copy 1 and Copy 2 represent the first copy and the second copy of the gene *dur3* in genomes of the remaining AOA. An empty cell indicates that the archaeon lacks the gene *dur3* or has only one copy of *dur3*.

| **Strain name** | **Source** | **Copy 1 *vs.* Ref 1** | **Copy 1 *vs.* Ref 2** | **Copy 2 *vs.* Ref 1** | **Copy 2 *vs.* Ref 2** |
| --- | --- | --- | --- | --- | --- |
| *Nitrosopumilus cobalaminigenes* HCA1 | marine | - | - | - | - |
| *Nitrosopumilus oxyclinae* HCE1 | marine | - | - | - | - |
| *Nitrosopumilus ureiphilus* PS0 | marine | 100% | 55.6% | 55.6% | 100% |
| *Nitrosopumilus zosterae* NM25 | marine | - | - | - | - |
| *Nitrosopumilus maritimus* SCM1 | marine | - | - | - | - |
| *Nitrosopumilus piranensis* D3C | marine | 91.5% | 54.0% | 55.9% | 86.4% |
| *Nitrosopumilus adriaticus* NF5 | marine | - | - | - | - |
| *Nitrosopumilus* sp. DDS1 | marine | - | - | - | - |
| *Candidatus* Nitrosopumilus koreensis AR1 | marine | 93.6% | 55.6% | 55.8% | 91.9% |
| *Candidatus* Nitrosopumilus sediminis AR2 | marine | 93.6% | 55.6% | 55.8% | 91.9% |
| *Nitrosopumilus* sp. SJ | marine | - | - | - | - |
| *Nitrosopumilus* sp. b2 | marine | 91.5% | 54.0% | 55.9% | 86.4% |
| *Nitrosopumilus* sp. b3 | marine | - | - | - | - |
| *Candidatus* Nitrosopumilus salaria BD31 | marine | - | - | - | - |
| *Nitrosopumilus* sp. YT1 | marine | - | - | - | - |
| Alpha AOA ma1 | marine | 89.2% | 63.0% | - | - |
| Alpha AOA ma8 | marine | - | - | - | - |
| *Candidatus* Nitrosopelagicus brevis CN25 | marine | - | - | - | - |
| *Candidatus* Nitrosopelagicus brevis U25 | marine | 71.2% | 50.9% | 57.8% | 61.9% |
| Gamma AOA mg1 | marine | 72.1% | 50.0% | - | - |
| Gamma AOA mg3 | marine | - | - | - | - |
| *Candidatus* Nitrosomarinus catalina SPOT01 | marine | 83.0% | 55.3% | - | - |
| *Candidatus* Cenarchaeum sp. HMK20 | marine | 81.5% | 56.1% | 55.7% | 75.7% |
| *Cenarchaeum symbiosum* A | marine | 81.7% | 54.3% | - | - |
| *Candidatus* Nitrosarchaeum limnium SFB1 | marine | - | - | - | - |
| *Candidatus* Nitrosarchaeum limnium BG20 | marine | - | - | - | - |
| *Nitrosarchaeum koreense* MY1 | terrestrial | - | - | - | - |
| *Nitrosoarchaeum* sp. AC2 | terrestrial | - | - | - | - |
| *Candidatus* Nitrosotenuis chungbukensis MY2 | terrestrial | - | - | - | - |
| *Candidatus* Nitrosotenuis cloacae SAT1 | terrestrial | 49.0% | 53.2% | - | - |
| *Candidatus* Nitrosotenuis sp. DW1 | terrestrial | - | - | - | - |
| *Candidatus* Nitrosotenuis uzonensis N4 | terrestrial | - | - | - | - |
| *Candidatus* Nitrosotenuis aquarius AQ6f | terrestrial | - | - | - | - |
| *Candidatus* Nitrosotalea devanaterra Nd1 | terrestrial | - | - | - | - |
| *Candidatus* Nitrosotalea sinensis Nd2 | terrestrial | - | - | - | - |
| *Candidatus* Nitrosotalea okcheonensis CS | terrestrial | 53.5% | 55.0% | 68.0% | 57.5% |
| *Nitrososphaera viennensis* EN76 | terrestrial | 59.0% | 61.2% | - | - |
| *Candidatus* Nitrososphaera gargensis Ga9.2 | terrestrial | 57.1% | 60.2% | - | - |
| *Candidatus* Nitrososphaera evergladensis SR1 | terrestrial | 58.2% | 59.7% | - | - |
| *Candidatus* Nitrosocosmicus oleophilus MY3 | terrestrial | 52.0% | 51.5% | - | - |
| *Candidatus* Nitrosocosmicus exaquare G61 | terrestrial | 52.0% | 52.2% | - | - |
| *Candidatus* Nitrosocosmicus franklandus C13 | terrestrial | 50.5% | 52.8% | - | - |
| *Candidatus* Nitrosocosmicus agrestis SS | terrestrial | 51.6% | 52.7% | - | - |
| *Candidatus* Nitrosocosmicus arcticus Kfb | terrestrial | 50.5% | 52.1% | - | - |
| *Candidatus* Nitrosocaldus cavascurensis SCU2 | terrestrial | 57.2% | 62.6% | - | - |
| *Candidatus* Nitrosocaldus islandicus 3F | terrestrial | 57.2% | 62.6% | - | - |

**References**

Abby, S.S., Melcher, M., Kerou, M., Krupovic, M., Stieglmeier, M., Rossel, C., et al. (2018). Candidatus Nitrosocaldus cavascurensis, an ammonia oxidizing, extremely thermophilic archaeon with a highly mobile genome. *Frontiers in Microbiology***,** 28.

Ahlgren, N.A., Chen, Y., Needham, D.M., Parada, A.E., Sachdeva, R., Trinh, V., et al. (2017). Genome and epigenome of a novel marine Thaumarchaeota strain suggest viral infection, phosphorothioation DNA modification and multiple restriction systems. *Environmental Microbiology* 19(6)**,** 2434-2452.

Alawi, M., Lipski, A., Sanders, T., and Spieck, E. (2007). Cultivation of a novel cold-adapted nitrite oxidizing betaproteobacterium from the Siberian Arctic. *The ISME Journal* 1(3)**,** 256-264.

Alves, R.J.E., Kerou, M., Zappe, A., Bittner, R., Abby, S.S., Schmidt, H.A., et al. (2019). Ammonia oxidation by the arctic terrestrial thaumarchaeote Candidatus Nitrosocosmicus arcticus is stimulated by increasing temperatures. *Frontiers in Microbiology* 10**,** 1571.

Bayer, B., Saito, M.A., McIlvin, M.R., Lücker, S., Moran, D.M., Lankiewicz, T.S., et al. (2021). Metabolic versatility of the nitrite-oxidizing bacterium Nitrospira marina and its proteomic response to oxygen-limited conditions. *The ISME Journal* 15(4)**,** 1025-1039.

Bayer, B., Vojvoda, J., Reinthaler, T., Reyes, C., Pinto, M., and Herndl, G.J. (2019). *Nitrosopumilus adriaticus* sp. nov. and *Nitrosopumilus piranensis* sp. nov., two ammonia-oxidizing archaea from the Adriatic Sea and members of the class *Nitrososphaeria*. *International Journal of Systematic and Evolutionary Microbiology* 69(7)**,** 1892-1902.

Blainey, P.C., Mosier, A.C., Potanina, A., Francis, C.A., and Quake, S.R. (2011). Genome of a low-salinity ammonia-oxidizing archaeon determined by single-cell and metagenomic analysis. *PloS One* 6(2)**,** e16626.

Bock, E., Sundermeyer-Klinger, H., and Stackebrandt, E. (1983). New facultative lithoautotrophic nitrite-oxidizing bacteria. *Archives of Microbiology* 136**,** 281-284.

Bollmann, A., Sedlacek, C.J., Norton, J., Laanbroek, H.J., Suwa, Y., Stein, L.Y., et al. (2013). Complete genome sequence of *Nitrosomonas* sp. Is79, an ammonia oxidizing bacterium adapted to low ammonium concentrations. *Standards in Genomic Sciences* 7(3)**,** 469-482.

Campbell, M.A., Chain, P.S., Dang, H., El Sheikh, A.F., Norton, J.M., Ward, N.L., et al. (2011). Nitrosococcus watsonii sp. nov., a new species of marine obligate ammonia-oxidizing bacteria that is not omnipresent in the world's oceans: calls to validate the names ‘Nitrosococcus halophilus’ and ‘Nitrosomonas mobilis’. *FEMS Microbiology Ecology* 76(1)**,** 39-48.

Carini, P., Dupont, C.L., and Santoro, A.E. (2018). Patterns of thaumarchaeal gene expression in culture and diverse marine environments. *Environmental Microbiology* 20(6)**,** 2112-2124.

Chain, P., Lamerdin, J., Larimer, F., Regala, W., Lao, V., Land, M., et al. (2003). Complete genome sequence of the ammonia-oxidizing bacterium and obligate chemolithoautotroph Nitrosomonas europaea. *Journal of Bacteriology* 185(9)**,** 2759-2773.

Daebeler, A., Herbold, C.W., Vierheilig, J., Sedlacek, C.J., Pjevac, P., Albertsen, M., et al. (2018). Cultivation and genomic analysis of “*Candidatus* Nitrosocaldus islandicus,” an obligately thermophilic, ammonia-oxidizing thaumarchaeon from a hot spring biofilm in Graendalur Valley, Iceland. *Frontiers in Microbiology* 9**,** 193.

Daims, H., Lebedeva, E.V., Pjevac, P., Han, P., Herbold, C., Albertsen, M., et al. (2015). Complete nitrification by Nitrospira bacteria. *Nature* 528(7583)**,** 504-509.

Ehrich, S., Behrens, D., Lebedeva, E., Ludwig, W., and Bock, E. (1995). A new obligately chemolithoautotrophic, nitrite-oxidizing bacterium, *Nitrospira moscoviensis* sp. nov. and its phylogenetic relationship. *Archives of Microbiology* 164**,** 16-23.

Fujitani, H., Kumagai, A., Ushiki, N., Momiuchi, K., and Tsuneda, S. (2015). Selective isolation of ammonia-oxidizing bacteria from autotrophic nitrifying granules by applying cell-sorting and sub-culturing of microcolonies. *Frontiers in Microbiology* 6**,** 1159.

Fujitani, H., Momiuchi, K., Ishii, K., Nomachi, M., Kikuchi, S., Ushiki, N., et al. (2020). Genomic and physiological characteristics of a novel nitrite-oxidizing *Nitrospira* strain isolated from a drinking water treatment plant. *Frontiers in Microbiology* 11**,** 545190.

Hallam, S.J., Konstantinidis, K.T., Putnam, N., Schleper, C., Watanabe, Y.-i., Sugahara, J., et al. (2006). Genomic analysis of the uncultivated marine crenarchaeote *Cenarchaeum symbiosum*. *Proceedings of the National Academy of Sciences* 103(48)**,** 18296-18301.

Herbold, C.W., Lehtovirta‐Morley, L.E., Jung, M.Y., Jehmlich, N., Hausmann, B., Han, P., et al. (2017). Ammonia‐oxidising archaea living at low pH: insights from comparative genomics. *Environmental Microbiology* 19(12)**,** 4939-4952.

Jung, M.-Y., Park, S.-J., Kim, S.-J., Kim, J.-G., Sinninghe Damsté, J.S., Jeon, C.O., et al. (2014). A mesophilic, autotrophic, ammonia-oxidizing archaeon of thaumarchaeal group I. 1a cultivated from a deep oligotrophic soil horizon. *Applied and Environmental Microbiology* 80(12)**,** 3645-3655.

Jung, M.-Y., Park, S.-J., Min, D., Kim, J.-S., Rijpstra, W.I.C., Sinninghe Damsté, J.S., et al. (2011). Enrichment and characterization of an autotrophic ammonia-oxidizing archaeon of mesophilic crenarchaeal group I. 1a from an agricultural soil. *Applied and Environmental Microbiology* 77(24)**,** 8635-8647.

Jung, M.Y., Kim, J.G., Sinninghe Damsté, J.S., Rijpstra, W.I.C., Madsen, E.L., Kim, S.J., et al. (2016). A hydrophobic ammonia‐oxidizing archaeon of the *Nitrosocosmicus* clade isolated from coal tar‐contaminated sediment. *Environmental Microbiology Reports* 8(6)**,** 983-992.

Kerou, M., Offre, P., Valledor, L., Abby, S.S., Melcher, M., Nagler, M., et al. (2016). Proteomics and comparative genomics of Nitrososphaera viennensis reveal the core genome and adaptations of archaeal ammonia oxidizers. *Proceedings of the National Academy of Sciences* 113(49)**,** E7937-E7946.

Keuter, S., Koch, H., Sass, K., Wegen, S., Lee, N., Lücker, S., et al. (2022). Some like it cold: the cellular organization and physiological limits of cold‐tolerant nitrite‐oxidizing Nitrotoga. *Environmental Microbiology* 24(4)**,** 2059-2077.

Kim, B.K., Jung, M.-Y., Yu, D.S., Park, S.-J., Oh, T.K., Rhee, S.-K., et al. (2011). Genome sequence of an ammonia-oxidizing soil archaeon, “*Candidatus* Nitrosoarchaeum koreensis” MY1". Journal of bacteriology 193(19):5539-5540

Kim, J.-G., Park, S.-J., Sinninghe Damsté, J.S., Schouten, S., Rijpstra, W.I.C., Jung, M.-Y., et al. (2016). Hydrogen peroxide detoxification is a key mechanism for growth of ammonia-oxidizing archaea. *Proceedings of the National Academy of Sciences* 113(28)**,** 7888-7893.

Klotz, M.G., Arp, D.J., Chain, P.S., El-Sheikh, A.F., Hauser, L.J., Hommes, N.G., et al. (2006). Complete genome sequence of the marine, chemolithoautotrophic, ammonia-oxidizing bacterium *Nitrosococcus oceani* ATCC 19707. *Applied and Environmental Microbiology* 72(9)**,** 6299-6315.

Koch, H., Lücker, S., Albertsen, M., Kitzinger, K., Herbold, C., Spieck, E., et al. (2015). Expanded metabolic versatility of ubiquitous nitrite-oxidizing bacteria from the genus *Nitrospira*. *Proceedings of the National Academy of Sciences* 112(36)**,** 11371-11376.

Könneke, M., Bernhard, A.E., de La Torre, J.R., Walker, C.B., Waterbury, J.B., and Stahl, D.A. (2005). Isolation of an autotrophic ammonia-oxidizing marine archaeon. *Nature* 437(7058)**,** 543-546.

Koops, H.-P., Böttcher, B., Möller, U., Pommerening-Röser, A., and Stehr, G. (1990). Description of a new species of Nitrosococcus. *Archives of Microbiology* 154(3)**,** 244-248.

Kozlowski, J.A., Kits, K.D., and Stein, L.Y. (2016). Complete genome sequence of *Nitrosomonas ureae* strain Nm10, an Oligotrophic Group 6a *Nitrosomonad*. *Genome Announcements* 4(2)**,** e00094-00016.

Lebedeva, E.V., Hatzenpichler, R., Pelletier, E., Schuster, N., Hauzmayer, S., Bulaev, A., et al. (2013). Enrichment and genome sequence of the group I. 1a ammonia-oxidizing archaeon “*Ca.* Nitrosotenuis uzonensis” representing a clade globally distributed in thermal habitats. *PLos One* 8(11)**,** e80835.

Lehtovirta-Morley, L.E., Ge, C., Ross, J., Yao, H., Nicol, G.W., and Prosser, J.I. (2014). Characterisation of terrestrial acidophilic archaeal ammonia oxidisers and their inhibition and stimulation by organic compounds. *FEMS Microbiology Ecology* 89(3)**,** 542-552.

Lehtovirta-Morley, L.E., Ross, J., Hink, L., Weber, E.B., Gubry-Rangin, C., Thion, C., et al. (2016). Isolation of ‘*Candidatus* Nitrosocosmicus franklandus’, a novel ureolytic soil archaeal ammonia oxidiser with tolerance to high ammonia concentration. *FEMS Microbiology Ecology* 92(5).

Lehtovirta-Morley, L.E., Stoecker, K., Vilcinskas, A., Prosser, J.I., and Nicol, G.W. (2011). Cultivation of an obligate acidophilic ammonia oxidizer from a nitrifying acid soil. *Proceedings of the National Academy of Sciences* 108(38)**,** 15892-15897.

Lewis, R., and Pramer, D. (1958). Isolation of Nitrosomonas in pure culture. *Journal of Bacteriology* 76(5)**,** 524-528.

Li, Y., Ding, K., Wen, X., Zhang, B., Shen, B., and Yang, Y. (2016). A novel ammonia-oxidizing archaeon from wastewater treatment plant: its enrichment, physiological and genomic characteristics. *Scientific Reports* 6(1)**,** 1-11.

Liu, L., Liu, M., Jiang, Y., Lin, W., and Luo, J. (2019). Physiological and genomic analysis of “*Candidatus* Nitrosocosmicus agrestis”, an ammonia tolerant ammonia-oxidizing archaeon from vegetable soil. *BioRxiv*.

Lücker, S., Nowka, B., Rattei, T., Spieck, E., and Daims, H. (2013). The genome of Nitrospina gracilis illuminates the metabolism and evolution of the major marine nitrite oxidizer. *Frontiers in Microbiology* 4**,** 27.

Lücker, S., Wagner, M., Maixner, F., Pelletier, E., Koch, H., Vacherie, B., et al. (2010). A Nitrospira metagenome illuminates the physiology and evolution of globally important nitrite-oxidizing bacteria. *Proceedings of the National Academy of Sciences* 107(30)**,** 13479-13484.

Matsutani, N., Nakagawa, T., Nakamura, K., Takahashi, R., Yoshihara, K., and Tokuyama, T. (2011). Enrichment of a novel marine ammonia-oxidizing archaeon obtained from sand of an eelgrass zone. *Microbes and Environments* 26(1)**,** 23-29.

Mosier, A.C., Allen, E.E., Kim, M., Ferriera, S., and Francis, C.A. (2012a). Genome sequence of “*Candidatus* Nitrosoarchaeum limnia” BG20, a low-salinity ammonia-oxidizing archaeon from the San Francisco Bay estuary. *Journal of Bacteriology* 194 (8), 2119-2120

Mosier, A.C., Allen, E.E., Kim, M., Ferriera, S., and Francis, C.A. (2012b). "Genome sequence of *Candidatus* Nitrosopumilus salaria” BD31, an ammonia-oxidizing archaeon from the San Francisco Bay estuary. *Journal of Bacteriology* 194 (8), 2121-2122

Mosier, A.C., Lund, M.B., and Francis, C.A. (2012c). Ecophysiology of an ammonia-oxidizing archaeon adapted to low-salinity habitats. *Microbial Ecology* 64(4)**,** 955-963.

Mueller, A.J., Jung, M.-Y., Strachan, C.R., Herbold, C.W., Kirkegaard, R.H., Wagner, M., et al. (2021). Genomic and kinetic analysis of novel *Nitrospinae* enriched by cell sorting. *The ISME Journal* 15(3)**,** 732-745.

Nicol, G.W., Hink, L., Gubry-Rangin, C., Prosser, J.I., and Lehtovirta-Morley, L.E. (2019). Genome Sequence of “*Candidatus* Nitrosocosmicus franklandus” C13, a terrestrial ammonia-oxidizing archaeon. *Microbiology Resource Announcements* 8(40)**,** e00435-00419.

Park, S.-J., Ghai, R., Martín-Cuadrado, A.-B., Rodríguez-Valera, F., Chung, W.-H., Kwon, K., et al. (2014). Genomes of two new ammonia-oxidizing archaea enriched from deep marine sediments. *PLoS One* 9(5)**,** e96449.

Picone, N., Pol, A., Mesman, R., van Kessel, M.A., Cremers, G., van Gelder, A.H., et al. (2021). Ammonia oxidation at pH 2.5 by a new gammaproteobacterial ammonia-oxidizing bacterium. *The ISME Journal* 15(4)**,** 1150-1164.

Qin, W., Heal, K.R., Ramdasi, R., Kobelt, J.N., Martens-Habbena, W., Bertagnolli, A.D., et al. (2017). Nitrosopumilus maritimus gen. nov., sp. nov., *Nitrosopumilus cobalaminigenes* sp. nov., *Nitrosopumilus oxyclinae* sp. nov., and Nitrosopumilus ureiphilus sp. nov., four marine ammonia-oxidizing archaea of the phylum Thaumarchaeota. *International Journal of Systematic and Evolutionary Microbiology* 67(12)**,** 5067-5079.

Qin, W., Zheng, Y., Zhao, F., Wang, Y., Urakawa, H., Martens-Habbena, W., et al. (2020). Alternative strategies of nutrient acquisition and energy conservation map to the biogeography of marine ammonia-oxidizing archaea. *The ISME Journal* 14(10)**,** 2595-2609.

Sakoula, D., Koch, H., Frank, J., Jetten, M.S., van Kessel, M.A., and Lücker, S. (2021). Enrichment and physiological characterization of a novel comammox *Nitrospira* indicates ammonium inhibition of complete nitrification. *The ISME Journal* 15(4)**,** 1010-1024.

Sakoula, D., Nowka, B., Spieck, E., Daims, H., and Lücker, S. (2018). The draft genome sequence of “*Nitrospira lenta*” strain BS10, a nitrite oxidizing bacterium isolated from activated sludge. *Standards in Genomic Sciences* 13(1)**,** 1-8.

Santoro, A.E., Dupont, C.L., Richter, R.A., Craig, M.T., Carini, P., McIlvin, M.R., et al. (2015). Genomic and proteomic characterization of “*Candidatus* Nitrosopelagicus brevis”: an ammonia-oxidizing archaeon from the open ocean. *Proceedings of the National Academy of Sciences* 112(4)**,** 1173-1178.

Sauder, L.A., Albertsen, M., Engel, K., Schwarz, J., Nielsen, P.H., Wagner, M., et al. (2017). Cultivation and characterization of *Candidatus* Nitrosocosmicus exaquare, an ammonia-oxidizing archaeon from a municipal wastewater treatment system. *The ISME Journal* 11(5)**,** 1142-1157.

Sauder, L.A., Engel, K., Lo, C.-C., Chain, P., and Neufeld, J.D. (2018). “*Candidatus* Nitrosotenuis aquarius,” an ammonia-oxidizing archaeon from a freshwater aquarium biofilter. *Applied and Environmental Microbiology* 84(19)**,** e01430-01418.

Spang, A., Poehlein, A., Offre, P., Zumbrägel, S., Haider, S., Rychlik, N., et al. (2012). The genome of the ammonia‐oxidizing *Candidatus* Nitrososphaera gargensis: insights into metabolic versatility and environmental adaptations. *Environmental Microbiology* 14(12)**,** 3122-3145.

Spieck, E., Hartwig, C., McCormack, I., Maixner, F., Wagner, M., Lipski, A., et al. (2006). Selective enrichment and molecular characterization of a previously uncultured Nitrospira‐like bacterium from activated sludge. *Environmental Microbiology* 8(3)**,** 405-415.

Starkenburg, S.R., Chain, P.S., Sayavedra-Soto, L.A., Hauser, L., Land, M.L., Larimer, F.W., et al. (2006). Genome sequence of the chemolithoautotrophic nitrite-oxidizing bacterium Nitrobacter winogradskyi Nb-255. *Applied and Environmental Microbiology* 72(3)**,** 2050-2063.

Starkenburg, S.R., Larimer, F.W., Stein, L.Y., Klotz, M.G., Chain, P.S., Sayavedra-Soto, L.A., et al. (2008). Complete genome sequence of *Nitrobacter hamburgensis* X14 and comparative genomic analysis of species within the genus Nitrobacter. *Applied and Environmental Microbiology* 74(9)**,** 2852-2863.

Stein, L.Y., Arp, D.J., Berube, P.M., Chain, P.S., Hauser, L., Jetten, M.S., et al. (2007). Whole‐genome analysis of the ammonia‐oxidizing bacterium, *Nitrosomonas eutropha* C91: implications for niche adaptation. *Environmental Microbiology* 9(12)**,** 2993-3007.

Stieglmeier, M., Klingl, A., Alves, R.J., Simon, K.-M.R., Melcher, M., Leisch, N., et al. (2014). *Nitrososphaera viennensis* gen. nov., sp. nov., an aerobic and mesophilic, ammonia-oxidizing archaeon from soil and a member of the archaeal phylum Thaumarchaeota. *International Journal of Systematic and Evolutionary Microbiology* 64(8)**,** 2738.

Suwa, Y., Sumino, T., and Noto, K. (1997). Phylogenetic relationships of activated sludge isolates of ammonia oxidizers with different sensitivities to ammonium sulfate. *The Journal of General and Applied Microbiology* 43(6)**,** 373-379.

Thandar, S.M., Ushiki, N., Fujitani, H., Sekiguchi, Y., and Tsuneda, S. (2016). Ecophysiology and comparative genomics of *Nitrosomonas mobilis* Ms1 isolated from autotrophic nitrifying granules of wastewater treatment bioreactor. *Frontiers in Microbiology* 7**,** 1869.

Ushiki, N., Fujitani, H., Shimada, Y., Morohoshi, T., Sekiguchi, Y., and Tsuneda, S. (2018). Genomic analysis of two phylogenetically distinct *Nitrospira* species reveals their genomic plasticity and functional diversity. *Frontiers in Microbiology* 8**,** 2637.

Walker, C.B., De La Torre, J., Klotz, M., Urakawa, H., Pinel, N., Arp, D., et al. (2010). *Nitrosopumilus maritimus* genome reveals unique mechanisms for nitrification and autotrophy in globally distributed marine crenarchaea. *Proceedings of the National Academy of Sciences* 107(19)**,** 8818-8823.

Wang, Y., Huang, J.M., Cui, G.J., Nunoura, T., Takaki, Y., Li, W.L., et al. (2019). Genomics insights into ecotype formation of ammonia‐oxidizing archaea in the deep ocean. *Environmental Microbiology* 21(2)**,** 716-729.

Watson, S.W. (1965). Characteristics of a marine nitrifying bacterium, *Nitrosocystis oceanus* sp. N.^1^. *Limnology and Oceanography* 10(suppl)**,** R274-R289.

Watson, S.W., and Waterbury, J.B. (1971). Characteristics of two marine nitrite oxidizing bacteria, Nitrospina gracilis nov. gen. nov. sp. and Nitrococcus mobilis nov. gen. nov. sp. *Archiv für Mikrobiologie* 77(3)**,** 203-230.

Suwa, Y., Norton, J.M., Bollmann, A., Klotz, M.G., Stein, L.Y., Laanbroek, H.J., et al. (2011). Genome sequence of Nitrosomonas sp. strain AL212, an ammonia-oxidizing bacterium sensitive to high levels of ammonia. *Journal of Bacteriology* 183 (18), 5047-8

Zhalnina, K.V., Dias, R., Leonard, M.T., Dorr de Quadros, P., Camargo, F.A., Drew, J.C., et al. (2014). Genome sequence of *Candidatus* Nitrososphaera evergladensis from group I. 1b enriched from Everglades soil reveals novel genomic features of the ammonia-oxidizing archaea. *PLos One* 9(7)**,** e101648.
