# Supplementary material for: Genomic insight into strategy, interaction and evolution of nitrifiers in metabolizing key labile-dissolved organic nitrogen in different environmental niches
Source: Front Microbiol. 2023 Dec 13;14:1273211. doi: 10.3389/fmicb.2023.1273211 (PMC10753782; doi:10.3389/fmicb.2023.1273211)
Supplement: Supplementary file 2 [file Presentation_1.pptx]

## Slide 1
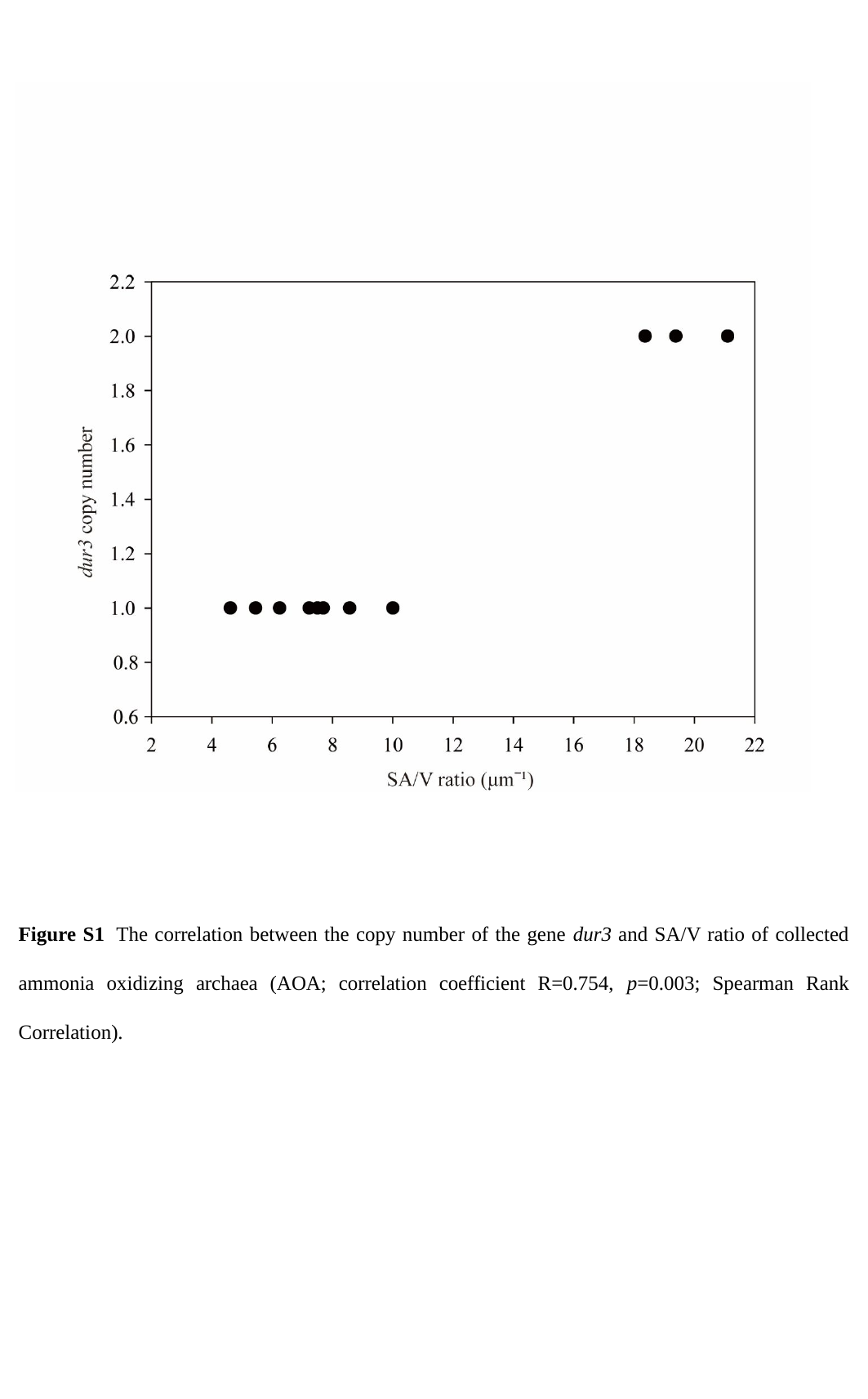

Figure S1  The correlation between the copy number of the gene dur3 and SA/V ratio of collected ammonia oxidizing archaea (AOA; correlation coefficient R=0.754, p=0.003; Spearman Rank Correlation).

## Slide 2
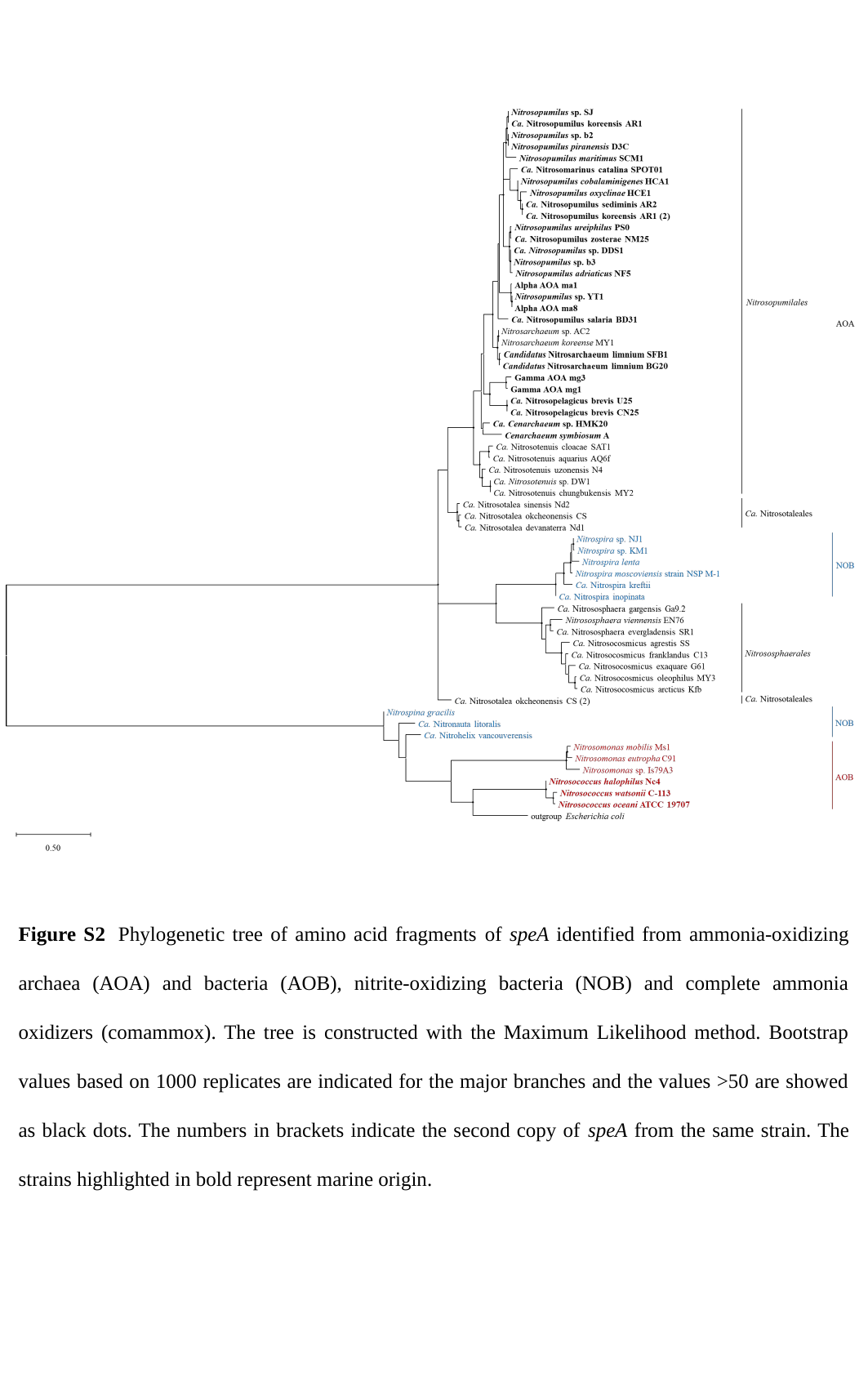

Figure S2  Phylogenetic tree of amino acid fragments of speA identified from ammonia-oxidizing archaea (AOA) and bacteria (AOB), nitrite-oxidizing bacteria (NOB) and complete ammonia oxidizers (comammox). The tree is constructed with the Maximum Likelihood method. Bootstrap values based on 1000 replicates are indicated for the major branches and the values >50 are showed as black dots. The numbers in brackets indicate the second copy of speA from the same strain. The strains highlighted in bold represent marine origin.
